# Topology only pre-training: towards generalised multi-domain graph models

**DOI:** 10.1007/s10618-026-01210-1

**Published:** 2026-05-09

**Authors:** Alex O. Davies, Riku Green, Telmo M. Silva Filho, Nirav Ajmeri

**Affiliations:** 1 School of Computer Science, University of Bristol, Bristol, UK; 2 School of Engineering Mathematics, University of Bristol, Bristol, UK

**Keywords:** Graph neural network, Graph representation learning, Graph foundation model

## Abstract

The principal benefit of unsupervised representation learning is that a pre-trained model can be fine-tuned where data or labels are scarce. Existing approaches for graph representation learning are domain specific, maintaining consistent node and edge features across the pre-training and target datasets. This has precluded transfer to multiple domains. We present Topology Only Pre-Training (ToP), a graph pre-training method based on node and edge feature exclusion. We show positive transfer on evaluation datasets from multiple domains, including domains not present in pre-training data, running directly contrary to assumptions made in contemporary works. On 75% of experiments, ToP models perform significantly ($P \le 0.01$) better than a supervised baseline. Performance is significantly positive on 85.7% of tasks when node and edge features are used in fine-tuning. We further show that out-of-domain topologies can produce more useful pre-training than in-domain. Under ToP we show better transfer from non-molecule pre-training, compared to molecule pre-training, on 79% of molecular benchmarks. Against the limited set of other generalist graph models ToP performs strongly, including against models with many orders of magnitude larger. These findings show that ToP opens broad areas of research in both transfer learning on scarcely populated graph domains and in graph foundation models.

**Supplementary Information:**

The online version contains supplementary material available at 10.1007/s10618-026-01210-1.

## Introduction

The pre-training and transfer paradigm, common for rich data such as images and text, allows strong performance where it might otherwise be lacking (Le-Khac et al. 2020; Devlin et al. 2018). Graph methods extend this idea to structured data, allowing much work on unsupervised learning over large datasets of graphs from a given domain (Chen et al. 2020; Khoshraftar and An 2024). Graph pre-training methods therefore allow good performance where raw data is abundant but labelled data is not, always assuming a consistent domain.

In other domains, such as images and text, it is common to pre-train on a large amalgamated dataset of samples, aiming to maximise coverage of the space (Chen et al. 2020; Touvron 2023; Ray 2023). Here data is not domain-specific, meaning that downstream tasks do not need to match the domain of pre-training data. Indeed large models pre-trained on these massive datasets show strong performance on downstream tasks even when the downstream domain is not present in the pre-training data. Models with this combination of size, data and performance are commonly referred to as *foundation models* (Myers et al. 2024).

There are substantial obstacles to taking this approach for graph data, the largest being feature heterogeneity between domains. Images or text from multiple domains are fundamentally the same data-type, uniform-size arrays of pixel values or sequences of tokens respectively, meaning that the multi-domain problem can be tackled head-on through data and model scale. Graphs, on the other hand, have a large diversity of possible feature sets. How a single model can usefully express highly heterogenous feature sets, for example both a text biography and the atomic number of an atom, is an open and challenging problem (Guo et al. 2019).

This has lead current research to argue or assume, but not show, that multi-domain pre-training on graphs is not feasible (Liu et al. 2023; Shirzad et al. 2022). As a consequence there are few works attempting learning across multiple domains of graphs. Some very recent works have proposed approaches using LLMs as feature transformations (Xu et al. 2025; Liu et al. 2023a), effectively moving a source domain into a unified text-featured domain. This drastically increases computational expense, and when features are not easily expressible as text, for example continuous features, LLM approaches presumably cannot be used.

The lack of a generalised pre-training method, which allows domain features to be included downstream, excludes areas of data sparsity or scarcity from the benefits of pre-training. Even on areas where data is plentiful enough for pre-training, it requires a different pre-trained model to be used each time, increasing the time, complexity, and cost of usage. Multi-domain pre-training would avoid these issues, both opening transfer learning as an option for domains with limited access to data, and providing a baseline model on which applications can be built.

### Pre-training without features

Graph learning can rely on information from both structure and features, with some coming only from structure. Assuming some shared signal in the structure between domains, therefore, beneficial multi-domain learning can occur during pre-training with features excluded. Motivated by this view we introduce **Topology** **Only** **Pre-Training (ToP)**, a generalised pre-training method for graph models. Under ToP pre-training is conducted with node and edge features excluded, but during downstream application they can be re-included by swapping components of a given model.

We investigate ToP as a pre-training method for graph data that is not limited to a given domain or task. The result of such pre-training is a model that can be transferred with performance increases on arbitrary graph-level tasks and domains. Experimentally we introduce and test two hypotheses: 

#### Hypothesis 1

 Excluding Features

Pre-training on multiple domains with node and edge features excluded can lead to consistent positive transfer compared to fully-supervised, task-specific models.

#### Hypothesis 2

Sample Diversity

With the exclusion of node and edge features out-of-domain pre-training can have greater benefits than in-domain pre-training across downstream tasks.

*Findings* We evaluate ToP using graph constrastive learning with both random through GraphCL (You et al. 2020) and adversarial augmentations though AD-GCL (Suresh et al. 2021). This evaluation includes the construction of an amalgamated, multi-domain dataset, spanning from road networks to molecules. We pre-train multiple models on different subsets of this amalgamated dataset and with varied augmentation schemes. We perform multiple fine-tuning runs for each model in order to show statistical significance for our experiments. Through this evaluation we contribute the following empirical findings:

1. *Effective pre-training.* Pre-training on varied domains, under the exclusion of node and edge features, leads to positive transfer. We find that under fine-tuning, results are at worst on-par, and on 76% of tasks significantly better, than a non-pre-trained baseline.
2. *Increased performance from domain diversity.* Pre-training without features from varied non-target domains, instead of the target domain, can bring greater downstream performance benefits. In the absence of features in pre-training a non-molecule learner consistently produces positive transfer compared to a molecule learner. On 56% of tasks the non-molecule model performs significantly better, and never significantly worse, than the molecule-only model.

**Contributions** We present two main contributions, although the work contains several more. Our first contribution is ToP, a novel multi-domain pre-training method for graph models. ToP is the first method that, to our knowledge, has the following desirable properties:

1. Positive transfer across multiple, highly diverse domains
2. Original node and edge features during transfer
3. No very large auxiliary models
4. No downstream feature transformations

Across molecular benchmarks our implementation of ToP out-performs benchmarks from the limited set of generalist GNN approaches that exist. ToP performs better-than or on-par with LLMs many orders of magnitude larger in both parameters and training dataset size. This provides great utility when pre-training data is not abundant.

Our second contribution is a new research direction: with domain features excluded, diverse non-domain pre-training can be more valuable than in-domain. This runs contrary to the single-domain training paradigm, and directly opposite to the assumptions made in other works (Mao et al. 2024; Shirzad et al. 2022). In Mao et al. (2024)’s review of LLMs for multi-domain graph models, enumerating why they show promise, it is assumed that *“...training on [graphs from multiple domains] simultaneously shows no positive transfer benefit while increasing the risk of the negative transfer”*. Our work shows that the opposite is true when pre-training is conducted only on structure. This implies that greater performance through ToP can be achieved simply by increasing the scale and diversity of the pre-training dataset.

We present several other secondary contributions. These include our multi-domain pre-training dataset, the first to span such a wide range of graph domains, from citation networks to neural connectomes and molecules. We demonstrate that models pre-trained with ToP gain a bias towards structural information, bolstering performance. Further, ToP models do this while properly utilising feature information.

*Assumptions and Theory* Multi-domain graph learning presents fundamentally different theoretical challenges than computer vision or natural language processing. Unlike uniform data types such as pixel arrays or token sequences, graphs exhibit heterogeneous feature spaces across domains that preclude straightforward theoretical analysis. The diversity of possible graph structures, feature types, and task objectives makes it mathematically intractable to prove a priori which structural patterns will transfer beneficially to arbitrary downstream domains and tasks.

Given these challenges, we adopt the empirically-driven approach that has proven successful in other domains. Foundational transfer learning works in computer vision and natural language processing, including BERT (Devlin et al. 2018) and landmark CNN studies (Yosinski et al. 2014; Donahue et al. 2014), established their validity through downstream performance rather than theoretical guarantees. In these fields, theoretical understanding has consistently followed empirical breakthroughs (Zeiler and Fergus 2014; Clark et al. 2019), and complete theoretical characterization of transferable representations remains an active research area even for areas with mature foundation models.

We therefore validate TOP through comprehensive empirical evaluation across diverse domains and tasks, with the expectation that these results will inform future theoretical development. This approach is particularly well-suited to graph learning, where the space of possible structural patterns and their semantic meanings across domains is not yet well-characterized.

*Organisation* In Sect. 2, we describe the notation and concepts necessary to understand our method and provide an overview of related works. In Sect. 3, we describe our generalised pre-training method ToP, and our hypotheses about it as a method. In Sect. 4, we describe how we evaluate the hypotheses detailed above, and present the findings of those experiments. In Sect. 5, we discuss the implications of the results from these experiments, as well as list the assumptions we make and threats to validity. In Sect. 6, we conclude and suggest avenues for future research.

## Background and related work

Here we describe the concepts necessary to understand the rest of our method, as well as closely related works. The most critical concept here is that we pre-train with representation learning then fine-tune over downstream datasets.

*Representation Learning* In most contexts, representation learning is the process of forming useful numerical encodings of non-tabular input data through an ‘encoder’ (Bengio et al. 2013). Let $X \in \mathcal {X}$ represent a random variable *X* in the data space $\mathcal {X}$, and $Y \in \mathcal {Y}$ the same for the output space. Representation learning aims, given their joint distribution *P*(*X*, *Y*), to learn a mapping $X \rightarrow \hat{X}$ that maximises their mutual information $I(\hat{X};Y)$. In basic terms, representation learners aim to transfer some piece of rich data into a vector that usefully describes that data.

*Contrastive Learning (CL)* CL is a semi-supervised school of representation learning based on altering input samples (Le-Khac et al. 2020). Let $\mathcal {T}$ be a set of small transformations called ‘augmentations’, where $t \in \mathcal {T}: X \rightarrow X'$. These augmentations are designed to manipulate the input sample in a way non-destructive to the ‘useful’ information it represents. Augmentations can be understood as a method to increase the semantic coverage in the input space. Common choices for images, for example, are grey-scaling and rotations. Intuitively, the augmentation defines an invariant transformation for the encoder to learn. Given an encoder’s mapping $F(X_i) \rightarrow \hat{X_i}$, multiple augmentations of the same input sample should be as similar as possible. In simple terms, contrastive learners aim to represent slight variations on the same sample in similar ways, for example through cosine similarity. Contrastive learning’s main benefit as pre-training is that it does not require labelled samples.

*Graph Representation Learning*Hamilton (2020) provides a comprehensive summary of methods for graph representation learning. Traditional methods to encode graphs as vectors include graph statistics such as centrality measures and clustering coefficients. Expressive Graph Neural Networks (GNNs) models, through parameterised message-passing, can produce powerful representations of graphs. A number of GNN architectures have been proposed with the Graph Isomorphism Network (GIN) being the most expressive in structural learning contexts (You et al. 2020).

*Graph Contrastive Learning* Multiple studies exist for graph contrastive learning (Chu et al. 2021; Hafidi et al. 2022; Hassani and Khasahmadi 2020; Hu et al. 2020; You et al. 2020; Zhu et al. 2021). The archetypal graph contrastive learning formulation, Graph Contrastive Learning (GraphCL), is put forward by You et al. (2020). In GraphCL and many subsequent works, such augmentations are performed randomly using fixed parameters, i.e., *‘drop 20% of nodes’*. These augmentation strategies do not vary based on the graph-instance being augmented.

*Adversarial Graph Contrastive Learning* In this work we use an ADversarial Graph Contrastive Learning (AD-GCL) formulation proposed by Suresh et al. (2021). The quality of augmentations is critical for graph contrastive learning (You et al. 2020; Zhu et al. 2021). To maximise expressivity in these augmentations adversarial strategies have been proposed (Hassani and Khasahmadi 2020; Zhao et al. 2023). Suresh et al. (2021)’s method AD-GCL trains both an encoder and a ‘view-learner’ $\mathcal {T}_{\theta }$. $\mathcal {T_{\theta }}$ is a parameterised ($\theta$) set of functions, learning augmentations that minimise mutual information between the source and augmented graphs through edge dropping. The encoder, adversarially to this, learns to maximise shared information between the respective augmentations. The updates for $\mathcal {T_{\theta }}$ are regularised such that augmentations are not entirely destructive. In their extensive experiments they demonstrate significant performance benefits over GraphCL (You et al. 2020).

*Graph Constrastive Coding* A similar work to ToP is Graph Contrastive Coding (GCC) (Qiu et al. 2020). As in this work, GCC’s primary aim is to sidestep the issue of varied feature sets between graph domains. GCC begins by generating subgraphs from various large graphs through ego sampling. As augmentations it used random walks with restarts, with embeddings of the seed node used for its contrastive loss, resulting in a local-focus model. This is in contrast to this work, where we focus on individual graph level representations, instead of pre-training as a route to tasks on large graphs. Also in contrast to our work, GCC does not provide a method for feature inclusion downstream, instead maintaining their use of positional encodings.

*LLMs on Graphs* Recently Large Language Models (LLMs) have been applied to building Graph Foundation Models (GFMs), with some arguing LLMs themselves may inherently be GFMs (Mao et al. 2024). While offering one-shot or few-shot application benefits, LLM size significantly increases graph model cost and complexity. Approaches include encoding graph structures in natural language (though schema choice impacts performance) (Fatemi et al. 2024), using LLMs as encoders to unify feature spaces across domains (requiring text-encodable, meaningful features and coherent embedding spaces) (Shang et al. 2024; Zhang et al. 2024; Xu et al. 2025; Liu et al. 2023a), and leveraging sequential descriptions like SMILES for molecular graphs (Liu et al. 2023b; Zhao et al. 2025; Cao et al. 2025). Models lacking explicit graph structure usage, such as InstructMol (Cao et al. 2025) and ChemDFM (Zhao et al. 2025), require substantially larger models and datasets to achieve comparable performance. A more detailed discussion of LLMs, LLMs on graphs, and LLMs in comparison to ToP, is provided in the Appendix.

## ToP: topology only pre-training

Assume a dataset with features $X \in \textbf{R}^{|V| \times D}$ and similar for edges, topology as an edgelist $E:\{ (v_1, v_2), \ldots \}$, and graph labels *y*, with graphs $G:\{X, E\}$. *y* is some target label. When a given model aims to learn some mapping $f(G) \rightarrow y$, in reality this is $f(X, E) \rightarrow y$. The usual graph learning assumption is that topology *E* and features *X* must be considered together for information to be useful. In other words their combination has mutual information with a target *y*, *I*(*y*; *X*, *E*).

We can assume that in the absence of features, structural information is still present, i.e. $I(y;E) \ge 0$. From this view, pre-training without features is well-motivated, with models learning generalised information about graph structures before features are introduced. This information from *E* is useful for downstream tasks, even while missing task-specific information from features. This is an abstraction away from feature-specific correlations, with abstraction a core quality of effective representation learners (Le-Khac et al. 2020). Removing features *X* necessarily removes their own information, but also the interdependence of features and structure:

1 $$\begin{aligned} I(y;E,X) \ge I(y;X) + I(y;E) \end{aligned}$$

In situations where data is abundant, therefore, pre-training on both *X* and *E* will likely still lead to higher performance.

Graphs without features are sampled from the same high dimensional space, which is highly constrained in real world graphs. As such they can be similar across highly varied domains, much as in the image space, even if they are entirely heterogenous with features included. In this case a wider range of topologies could span a wider set of structural patterns, and so contain more useful information, than a narrow range closer to a downstream domain.

This pre-training on only topologies would give an initial bias towards structural information during downstream transfer. Such a structural bias is encouraged in other works, in particular those works on Topological GNNs (Horn et al. 2021; Chen et al. 2021). Here layers or models themselves are designed to encourage learning from patterns in structure over patterns in features.

*Topology* *Only* *Pre-Training (ToP)* is a method for generalised pre-training of graph models. Under ToP pre-training is conducted only on topologies, with node and edge features replaced with the integer 1. This use of only topologies *G*, instead of graphs with specific features $\underline{G}$, allows pre-training on a multi-domain dataset. In the works prior to ToP, the use of such a graph dataset was not tractable (Liu et al. 2023; Shirzad et al. 2022; Zhang et al. 2023).

**Fig. 1:**
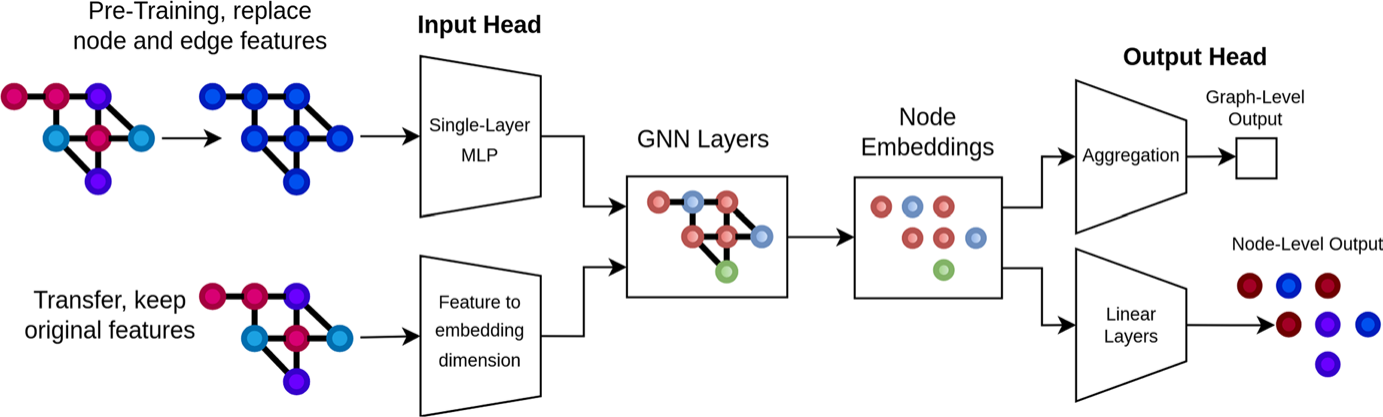
A schematic for ToP models. During pre-training, features are replaced with a single integer label for each node or edge (all identical), which are then passed to a single-layer MLP. This results in an identical input vector for each node and edge. During transfer, a different input head is used, moving the domain feature dimensions to the hidden dimensionality of the GNN encoder block. This allows arbitrary node and edge features to be included in transfer. Omitting the output head of the model allows transfer onto node and edge-level tasks

Figure 1 shows a schematic for ToP models. ToP’s implementation is elegant: Features are removed from nodes and edges and replaced with some arbitrary identical value. In our case all nodes and edges carry the integer value 1. The use of a uniform value ensures that all nodes carry the same feature vector on entry to GNN layers, which is equivalent to removing features entirely while still satisfying the input requirements of message-passing GNNs. With the input head some simple MLP, the choice of value has no significant impact, as the GNN layers are presented the same vector for all nodes and edges. An encoder is then pre-trained using some unsupervised representation learning method. In our implementation we use contrastive learning for pre-training, specifically AD-GCL, given its strong performance in representation learning on graphs.

By swapping the input head of the pre-trained model, arbitrary node and edge features can be included downstream, allowing integration of feature information alongside structural during transfer. In the same manner the output head of a model can be replaced or excluded entirely, allowing transfer onto node and edge-level tasks.

### Hypothesis 1: excluding features

*Pre-training on multiple domains with node and edge features excluded can lead to consistent and positive transfer compared to fully-supervised, task-specific models.* The motivating concept behind ToP is that as graph learning requires learning on both structural and feature levels, pre-training on only structure can lead to useful knowledge transfer. As such, a model that hasn’t undergone such pre-training should perform worse on downstream tasks when applied under the same supervised training conditions.

Supporting Hypothesis 1 means producing a model that has learnt useful representations of graphs *in general*. Our first step to achieving this is to construct a dataset that covers a variety of graph domains. Such a dataset should contain graphs that present a large diversity of multi-node patterns. Our approach is simple: include graphs from several domains and assume that there is adequate coverage of the multi-node patterns that occur in real-world graphs for a given set of downstream tasks. The same approach has been taken in the domains of text (Brown 2020) and images (Rombach et al. 2022), where enormous numbers of data samples are scraped from the web for generative pre-training. This relies on two assumptions 1) this dataset adequately covers the relevant space for downstream transfer, and 2) a single model can usefully represent such a space. These assumptions, as in other fields, are justified through performance metrics on downstream tasks on seen and unseen domains.

We formulate our test for Hypothesis 1, that pre-training on graphs without node or edge features is beneficial, as beating a simple supervised baseline. Said baseline is a model with the same architecture as the pre-trained model but without pre-training. Both models are then trained, or fine-tuned for the pre-trained model, on the same downstream datasets under the same conditions. On each downstream task, should the pre-trained model match or out-perform the supervised model, this represents positive transfer. Positive transfer on a task indicates that at least some of the learning during pre-training was beneficial to subsequent learning during fine-tuning. A positive transfer rate across datasets of more than 50% indicates that in-general transfer is positive, thus meeting the minimum criteria for Hypothesis 1. A high positive transfer rate strongly supports Hypothesis 1.

#### Hypothesis 2: Sample Diversity

*With the exclusion of node and edge features, out-of-domain pre-training can have greater performance benefits than in-domain pre-training across downstream tasks.* If structure-only learning is useful, as proposed by Hypothesis 1, it follows that out-of-domain of pre-training data with greater diversity may be more useful than in-domain. This relies on the assumption that a given encoder is learning to represent local node patterns, and as diverse data presents a wider range of such patterns, the model must learn more expressively how to represent such patterns.

From Hypothesis 2 we expect that for a given task models trained on a more diverse pre-training domain can bring better transfer than a model trained on the target domain. We do not argue that this is always true, only that in some settings out-of-domain samples can be more useful for pre-training than in-domain. To this end we compare two models, the first trained on a dataset from a single domain, as is the current practice. This single domain matches that of the downstream target dataset(s). The second model is instead trained on the rest of the data *while excluding the target domain*. The total size of the pre-training datasets for each model should match for fair comparison. By pre-training both models under the same controlled conditions (hyperparameters etc.), then evaluating their performance under fine-tuning, we directly compare pre-training on a single domain to pre-training on multiple domains. General positive transfer from the multi-domain model, in comparison to transfer from the in-domain model, would constitute a clear supporting evidence for Hypothesis 2.

### Models for evaluation

In order to address these hypotheses, we apply ToP over five principal different combinations of pre-training data and augmentation scheme. **GIN** An untrained GIN model of identical architecture to the other methods. This serves to benchmark what constitutes positive transfer. **ToP-Chem** An AD-GCL model fit only on molecules. This constitutes an in-domain model, albeit in the absence of domain features. **ToP-Social** An AD-GCL model fit only on non-molecules. This serves as a contrast to ToP-Chem, allowing us to assess the benefits of varied out-of-domain pre-training in comparison to in-domain pre-training. **ToP-All** An AD-GCL model fit on all the training data. This is our primary measure of the effectiveness of ToP. **ToP-All (CL)** A model trained across the whole amalgamated dataset with the loss function from You et al. (2020) using random edge dropping ($\lambda =0.2$). This allows us to assess the impact of adversarial augmentations in ToP pre-training.

Models during pre-training, unless specified, are six GIN (Xu et al. 2019) layers. Our pre-training setting is purely structural learning, and as a result, we require a GNN layer that is strongly expressive over graph structures. The GIN layer applies learnable transformations *after aggregation*, enabling strong discrimination of structural patterns as proposed in (Xu et al. 2019). They are maximally expressive among MPNNs, matching the 1-WL test, making it an ideal, and popular, choice for graph structure learning. We use single linear layers as input and output heads. The GIN model acts as our supervised baseline (Hypothesis 1 in Sect. 4). The ToP-Chem/ToP-Social models indicate the impact of training data diversity on downstream transfer performance (Hypothesis 2). The ToP-All (CL) model shows the impact of adversarial augmentations.

**Fig. 2:**
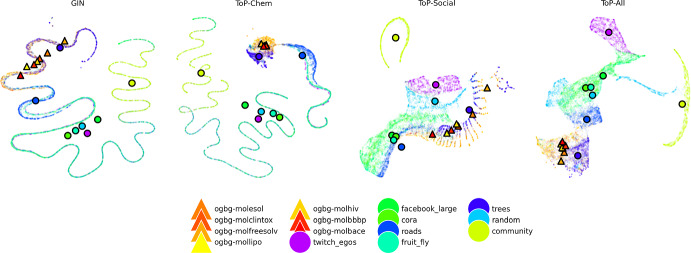
UMAP embeddings of encodings from each ToP model, as well as an untrained GIN model. Triangular markers show molecular graphs, and circles non-molecules. We plot the centroid of each dataset for added clarity

## Experiments

We employ AD-GCL (Suresh et al. 2021) as a contrastive pre-training framework. We take the downstream task on each validation set (see below) where available and add their relevant scores (MSE and $1-$ AUROC) as a crude monitor for the models’ representational abilities during pre-training. 5000 samples are used for non-molecular datasets. We conduct a limited Bayesian hyperparameter sweep. The heuristic used is addition of evaluation scores on each dataset weighted by the number of samples in that dataset. The aim here is to demonstrate that the hyperparameters found through the extensive testing in Suresh et al. (2021) are still appropriate for use in ToP. We find that the original parameters produce good performance with our crude monitor, so we use these for all pre-training runs. Specifically these hyperparameters are 100 epochs with a batch size of 512, learning rates of 0.001 for the encoder and view model, embedding and projection dimensions of 300 and a regularisation constant of 0.2.

While task-specific hyperparameter tuning could potentially improve results on individual benchmarks, we deliberately avoided this. Extensive per-task optimization would undermine our central claim: that a single pre-trained model with fixed parameters can provide positive transfer across diverse domains. Hyperparameter tuning using this metric, or another metric based on our specific datasets, biases towards this *set of domains* instead of *arbitrary new domains*. Our approach, using validated parameters from prior work, ensures fair comparison and demonstrates that ToP’s benefits arise from the pre-training strategy itself, not from hyperparameter optimization.

Figure 2 shows UMAP embeddings of encodings of the validation datasets for our models. Larger versions for the four ToP models can be found in the Appendix.

### Datasets

Evaluating ToP requires collecting datasets of graphs from multiple domains. A portion of these datasets should have downstream tasks, the useful information for which relies on both structure and features, with varied weighting to one or the other.

Our datasets are as-follows, with statistics provided in Appendix Table 4. We propose domain-relevant tasks for the Facebook and Roads datasets, and use existing tasks where available for our other datasets. molpcba is in itself a reasonably sized dataset for supervised learning, and as such not appropriate for our investigation of transfer learning on small datasets. As a result we employ 7 standard single-target molecular benchmarks (molesol, molfreesolv, mollipo, molclintox, molbbbp, molbace, molhiv) from MoleculeNet (Wu et al. 2017).

***Pre-Training Only***

- *molpcba* (Hu et al. 2020) A curated dataset of 437929 small molecules, from which we use 250000.
- *Cora* (McCallum et al. 2000) A much used citation graph.
- *Fly Brain* (Winding et al. 2023) The full neural connectome of a fruit fly larvae. As the original multi-graph is dense at 548000 edges, we include a single edge between neurons if there are more than two synapses between them.

***Pre-Training and Evaluation***

- *Facebook* (Rozemberczki et al. 2021) A single graph of page-page connections on Facebook. Used both in pre-training and downstream transfer. On a graph level the downstream task is predicting average clustering, and we also evaluate transfer to node classification. This task does not use feature information.
- *Twitch* (Rozemberczki et al. 2020) Ego networks from the streaming platform Twitch, used both in pre-training and downstream transfer, with a downstream binary classification task includd with the source dataset. This task does not use feature information.
- *Roads* (Leskovec et al. 2009) The road network of Pennsylvania. Used both in pre-training and downstream transfer, with the task set to be predicting graph diameter normalised by graph size. This task does not use feature information.

***Evaluation Only***

- *Benchmark Molecules* (Hu et al. 2020) molesol, molfreesolv, mollipo, molclintox, molbbbp, molbace, molhiv. Datasets sampled from MoleculeNet, with single-target downstream tasks. These are used only in downstream transfer. These benchmarks have pre-prepared node and edge features, allowing us to measure the extent to which ToP models can integrate feature information alongside structural. We source train/validation/test splits from the Open Graph Benchmark (Hu 2020).
- *Trees* Trees, of a randomly sampled maximum depth, and a randomly fixed probability of branching at each level, with a downstream task of predicting depth. Used only in downstream evaluation. This task does not use feature information.
- *Random* Erdos-Renyi (ER) graphs (Erdös and Rényi 2011), with a number of nodes and edge probability randomly sampled, and predicting edge probabilities as a downstream task. Used only in downstream evaluation. This task does not use feature information.
- *Community* Small ER subgraphs, with a set intra-subgraph and random inter-subgraph connection probability, with inter-subgraph connectivity as target. Used only in downstream evaluation. This task does not use feature information.

***Datasets from Single Graphs***

As the Facebook, Cora, Roads and Fly Brain datasets consist of a single graph, we employ exploration sampling to construct datasets of many smaller graphs. We use a randomly selected exploration sampler for each sample (Rozemberczki et al. 2020), which in turn samples a random number of nodes in the range $24 \le |V| \le 96$ from the source graph. The use of a selection of exploration samplers should further ensure that a large variety of graphs and structural patterns are included in the datasets.

**Table 1:** Scores for fine-tuning on each validation dataset. Molecular benchmarks here do not include node or edge features

	GIN	ToP-All(CL)	ToP-Chem	ToP-Social	ToP-All	
$\downarrow$ Facebook	0.31 ± 0.07	** 0.24** ±** 0.06**	1.17 ± 0.17	$\underline{\boldsymbol{0.13} \pm \boldsymbol{0.00}}$	$\underline{\boldsymbol{0.13} \pm \boldsymbol{0.00}}$	$\checkmark$
$\uparrow$ Twitch Egos	0.72 ± 0.01	** 0.74** ± ** 0.01**	0.69 ± 0.02	** 0.74** ± ** 0.01**	$\underline{\boldsymbol{0.79} \pm \boldsymbol{0.01}}$	$\checkmark$
$\downarrow$ Roads	0.46 ± 0.07	** 0.25** ± ** 0.03**	0.57 ± 0.07	$\underline{\boldsymbol{0.10} \pm \boldsymbol{0.00}}$	$\underline{\boldsymbol{0.10} \pm \boldsymbol{0.00}}$	$\checkmark$
$\downarrow$ Trees	0.20 ± 0.01	** 0.19** ± ** 0.02**	0.24 ± 0.03	$\underline{\boldsymbol{0.12} \pm \boldsymbol{0.00}}$	$\underline{\boldsymbol{0.12} \pm \boldsymbol{0.00}}$	$\checkmark$
$\downarrow$ Community	0.74 ± 0.11	** 0.31** ± ** 0.07**	** 0.72** ± ** 0.13**	** 0.02** ± 0.00	0.02 ± 0.00	$\checkmark$
$\downarrow$ Random	0.53 ± 0.23	** 0.29** ± ** 0.10**	0.59 ± 0.09	$\underline{\boldsymbol{0.04} \pm \boldsymbol{0.00}}$	$\underline{\boldsymbol{0.04} \pm \boldsymbol{0.00}}$	$\checkmark$
$\downarrow$ molfreesolv	4.98 ± 0.48	** 4.20** ± ** 0.10**	** 3.89** ± ** 0.21**	$\underline{\boldsymbol{3.83} \pm \boldsymbol{0.11}}$	4.26 ± 0.03	$\checkmark$
$\downarrow$ molesol	1.78 ± 0.09	** 1.57** ± ** 0.06**	** 1.44** ± ** 0.09**	** 1.47** ± ** 0.04**	$\underline{\boldsymbol{1.31}\pm \boldsymbol{0.03}}$	$\checkmark$
$\downarrow$ mollipo	1.13 ± 0.04	** 1.07**± ** 0.03**	** 1.04** ± ** 0.02**	** 0.99** ± ** 0.01**	$\underline{\boldsymbol{0.97} \pm \boldsymbol{0.01}}$	$\checkmark$
$\uparrow$ molclintox	0.53 ± 0.04	0.46 ± 0.03	0.51 ± 0.03	0.50 ± 0.05	$\underline{\boldsymbol{0.55} \pm \boldsymbol{0.11}}$	
$\uparrow$ molbbbp	0.57 ± 0.04	$\underline{\boldsymbol{0.60} \pm \boldsymbol{0.03}}$	0.54 ± 0.04	** 0.60** ± ** 0.03**	** 0.59** ± ** 0.02**	
$\uparrow$ molbace	0.68 ± 0.05	$\underline{\boldsymbol{0.76} \pm \boldsymbol{0.03}}$	0.63 ± 0.13	** 0.75** ± ** 0.03**	** 0.71** ± ** 0.02**	
$\uparrow$ molhiv	0.36 ± 0.06	** 0.50** ± ** 0.14**	** 0.54** ± ** 0.13**	** 0.64** ± ** 0.05**	$\underline{\boldsymbol{0.66} \pm \boldsymbol{0.04}}$	$\checkmark$
Best	0%	15.4%	0.0%	38.5%	69.2%	
Positive	–	92.3%	38.5%	92.3%	100%	

For the classification datasets we report AUROC ($\uparrow$) and for regression datasets RMSE ($\downarrow$).**Bold** text indicates a result beats the supervised (GIN) baseline. Underlined text indicates a superior result, down to three significant figures. Checkmarks $\checkmark$ indicate that the ToP-All model significantly out-performs the GIN baseline at $p < 0.01$. ‘Best’ indicates the proportion of datasets over which a model has superior performance, and ‘Positive’ the proportion of datasets over which performance is superior to the fully-supervised baseline

*Compute* Pre-training was conducted on a workstation with an NVIDIA A6000, using around 16GB of VRAM capacity. Pre-training the ToP-All model took a little more than eight hours. Transfer results were produced on consumer laptops and on GPU use around 3GB of video memory with batch sizes of 128.

### Transfer

We apply our pre-trained models to various downstream tasks through transfer learning. This follows stages of abstraction away from ToP pre-training: first we fine tune on datasets without features, then on datasets with features included, and then compare ToP models to reasonable benchmarks.

We replace the output head with a simple two-layer MLP, with the additional layer a single output node. We then perform 10 fine-tuning runs of 50 epochs on each validation dataset, with and without features, for each pre-trained model. Table 1 presents results of transfer and fine-tuning without features. We perform no hyperparameter optimisation here, in order to avoid introducing biases towards ToP over benchmarks, so maintain the same learning rate of 0.001 and batch sizes of 512. In contrast where external results are reported they generally follow a broad hyperparameter optimisation. 50 epochs is an upper-limit, and results are reported for the model at its lowest validation loss during these 50 epochs.

#### Features excluded

Here features are excluded during downstream transfer, mirroring the features presented during pre-training with ToP. The ToP-All model consistently out-performs the other models under fine-tuning on the majority of datasets. At $p \le 0.01$ the ToP-All model significantly out-performs the GIN baseline on 77% of datasets. When performance is not significantly better, it is never significantly worse. The standard deviation of the results on each dataset are lower than those for the non-pre-trained and domain-specific pre-trained models. In the Appendix we show validation loss on several datasets during fine-tuning for the ToP-All model and an un-pre-trained GIN with the same architecture.

On our evaluation-only datasets (Trees, Community, Random), on which no models are pre-trained, we see markedly better performance from models that are pre-trained on a range of domains. In particular the ToP-All and ToP-Social models drastically out-perform our supervised baseline and ToP-Chem. On the Trees dataset, which intuitively and on aggregate measures is the closest of the evaluation-only datasets to molecular data, ToP-Chem also has negative transfer.

Pre-training only on molecules (‘Chem’ data subsets) results at-best on-par transfer compared to the supervised GIN baseline, with negative transfer on the majority of datasets. Pre-training only on non-molecules, in comparison, results in positive transfer on the large majority of datasets and pre-training methods. At $p \le 0.01$ the ToP-Social model significantly out-performs the ToP-Chem model on the majority of datasets. As before, where performance is not significantly better, it is never significantly worse. These results corroborate the results presented in Table 1, with the Chem model consistently out-performed by non-molecular models. We find that under pre-training with random edge dropping performance actually drops when including molecular data (‘Social’ vs ‘All’). Using a variety of non-molecular graphs in-general provides a more solid pre-training foundation than including molecules for both methods of augmentation.

**Table 2:** Scores for fine-tuning pre-trained GIN models (and a non-pre-trained baseline) on the molecular evaluation datasets with node and edge features included via projection heads

	GIN	ToP-All(CL)	ToP-Chem	ToP-Social	ToP-All	
$\downarrow$ molfreesolv	4.00 ± 0.43	4.00 ± 0.24	4.16 ± 0.29	**3**.**61** ±**0**.**43**	$\underline{\boldsymbol{3.01} \pm \boldsymbol{0.59}}$	$\checkmark$
$\downarrow$ molesol	1.72 ± 0.25	**1**.**18** ±**0**.**09**	**1**.**62** ±**0**.**31**	**1**.**03** ± 0.17	$\underline{\boldsymbol{0.90}\pm \boldsymbol{0.04}}$	$\checkmark$
$\downarrow$ mollipo	1.09 ± 0.05	**0**.**99** ±**0**.**05**	1.09 ± 0.06	**0**.**80** ± 0.02	$\underline{\boldsymbol{0.79}\pm \boldsymbol{0.02}}$	$\checkmark$
$\uparrow$ molclintox	0.65 ± 0.12	0.56 ± 0.16	0.52 ± 0.03	$\underline{\boldsymbol{0.86} \pm \boldsymbol{0.02}}$	**0**.**84** ± 0.05	$\checkmark$
$\uparrow$ molbbbp	0.60 ± 0.03	**0**.**63** ±**0**.**04**	**0**.**61** ±**0**.**03**	**0**.**62** ± 0.03	$\underline{\boldsymbol{0.67} \pm \boldsymbol{0.02}}$	$\checkmark$
$\uparrow$ molbace	$\underline{0.75 \pm 0.03}$	0.72 ± **0**.**05**	0.70 ± 0.07	0.70 ± 0.05	0.72 ± 0.05	
$\uparrow$ molhiv	0.32 ± 0.03	**0**.**46** ± **0**.**14**	**0**.**37** ±**0**.**07**	$\underline{\boldsymbol{0.62} \pm \boldsymbol{0.11}}$	**0**.**57** ± 0.13	$\checkmark$
Best	14.3%	0.0%	0.0%	28.6%	57.1%	
Positive	–	57.1%	42.9%	85.7%	85.7%	

For the classification datasets we report AUROC ($\uparrow$) and for regression datasets RMSE ($\downarrow$). **Bold** text indicates a result beats the supervised (GIN) baseline. Underlined text indicates a superior result, down to three significant figures. Checkmarks $\checkmark$ indicate that the ToP-All model significantly out-performs the GIN baseline at $p < 0.01$. ‘Best’ indicates the proportion of datasets over which a model has superior performance, and ‘Positive’ the proportion of datasets over which performance is superior to the fully-supervised baseline

*Features Included* Here features are included for downstream transfer. We replace the input head of our model with a three layer Multi-Layer Perceptron (MLP) for both node and edge features, with the extra depth to allow greater expressivity over the feature space, then perform fine-tuning runs on our molecular datasets. Table 2 presents the results of this transfer and fine-tuning. These tasks are assumed to require node and edge features to obtain good performance, and here positive transfer rates are similar to when no features are included. This shows that the focus on structure during ToP pre-training does not inhibit the use of feature-based information in downstream transfer. The ToP-All and ToP-Social models again demonstrate consistent positive transfer, and on most datasets their performance improves compared to when features were excluded. Transfer is not significantly positive on only one dataset, molbace, where structural information is less useful to the specific task. We investigate this result further in Sect. 10.2, with the same results for varied GNN backbones presented in the Appendix.

### Comparison to other methods

The selection of valid benchmarks from other works is limited. We start by splitting existing works into two broad categories, generalists that function on any graph domain, and specialists that are designed for a specific domain of graph data. The selection of specialist graph models in chemistry is broad, but not necessarily a fair comparison, as these works do not share the same fundamental aims as ToP. They are something of a best-case for pre-training: abundant pre-training data, without missing features. In the following sections we include specialist GNNs, all pre-trained with contrastive learning, as well as specialist LLMs designed for molecular tasks. We don’t expect ToP models to out-perform these specialists. Instead, we include these results to evaluate how our multi-domain pre-training framework compares to the current state of the art within a domain.

The selection of generalist models is far more limited. GCC is, to the best of the author’s knowledge, the only other generalist GNN work that does not rely on some level of feature transformation using LLMs. This carries the significant caveat that the original work completely eliminates features, and the information they carry, from downstream transfer. We extend the original GCC work somewhat, swapping the input and output heads as with our own models, in order to provide fair comparison against ToP. Explicitly, GCC does not include domain features; feature inclusion with GCC is an extension using techniques from this work.

In Table 3 we compare ToP to other pre-training schemes across molecular tasks. Results are sourced from the original works, where they typically follow an extensive and task-specific hyperparameter tuning process. These methods are divided into specialist (domain-specific) and generalist (domain-agnostic) approaches. For domain-specific contrastive GNN approaches we include InfoGraph (Xu et al. 2021), GraphCL (Hassani and Khasahmadi 2020) and AD-GCL (Suresh et al. 2021). These models are pre-trained with all features included, and hyper-parameters optimised, over Zinc (2 M). We also include MolXPT (Liu et al. 2023b), InstructMol (Cao et al. 2025) and ChemDFM (Zhao et al. 2025), chemistry-specific LLM approaches.

**Table 3:** Our results for the ToP-All and ToP-Social models presented alongside the same results from other pre-training works

	clintox (1329)	bbbp (1835)	bace (1361)	hiv (37014)
*Specialist GNN*				
InfoGraph (2.4M)	0.70 ± 0.03	0.69 ± 0.01	0.76 ± 0.02	0.76 ± 0.02
GraphCL (2.4M)	0.76 ± 0.03	**0**.**70** ± **0**.**01**	0.75 ± 0.01	**0**.**78** ± **0**.**01**
AD-GCL (2.4M)	**0**.**80** ±**0**.**04**	**0**.**70** ±**0**.**01**	**0**.**79** ±**0**.**01**	**0**.**78** ±**0**.**01**
*Specialist LLM*				
MolXPT (350 M)	**0**.**95** ±**0**.**00**	**0**.**80** ±**0**.**01**	**0**.**88** ±**0**.**01**	**0**.**78** ±**0**.**00**
InstructMol (7B)		0.70	0.82	0.69
ChemDFM (13B)	0.90	0.67	0.78	0.74
*Generalist LLM*				
GPT-4 ($>200B^{1}$)	0.52	**0**.62	0.63	0.66
LLaMa-2 (13B)	0.46	0.60	0.26	0.29
Galactica (30B) $\dagger$	**0**.**82**	0.60	**0**.**73**	**0**.**76**
*Generalist GNN*				
GCC (untrained) (200k)	0.50 ± 0.03	0.59 ± 0.01	0.44 ± 0.04	0.46 ± 0.01
GCC (200k)	0.40 ± 0.07	0.52 ± 0.02	0.50 ± 0.04	0.52 ± 0.01
GCC+Feat (untrained)	0.79 ± 0.04	0.64 ± 0.02	0.70 ± 0.05	**0**.**70** ±**0**.**03**
GCC+Feat	0.50 ± 0.04	0.61 ± 0.03	0.70 ± 0.1	0.68 ± 0.05
ToP-**Social** (2.4M)	**0**.**86** ±**0**.**02**	0.62 ± 0.03	0.70 ± 0.05	0.62 ± 0.11
ToP-**All**	0.84 ± 0.05	**0**.**67** ±**0**.**02**	**0**.**72** ±**0**.**05**	0.57 ± 0.13

Alongside dataset names we include the total size of the dataset. Results are ROC-AUC, and other than for ToP and GCC, taken verbatim from the respective work. Where standard deviations or errors are not present, they were also not present in the respective work. (*n*B) indicates *x* billion parameters. Bold text indicates a superior result within a type of model

Best-guess speculation, as OpenAI is closed-source

We also include results from other domain-agnostic graph models. For LLM-based approaches on these tasks results are sourced the ChemDFM work (Zhao et al. 2025). Performing molecular tasks with these models relies on the same assumptions as chemistry-specific models, but without fine-tuning on chemical literature.^1^

**Fig. 3:**
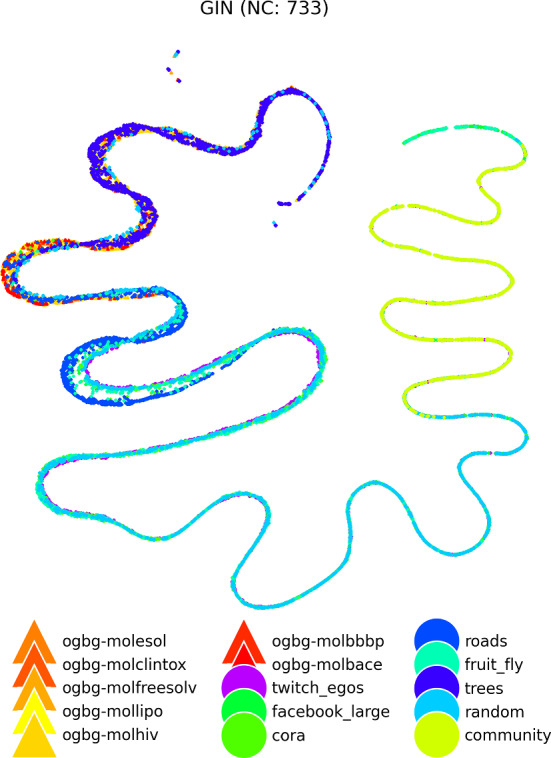
A UMAP embedding of encodings from an untrained encoder

Finally we include GCC, to our knowledge the only other framework or approach for producing generalised graph models without relying on LLMs, albeit at the cost of feature inclusion for downstream tasks. We fine-tune the two pre-trained encoders packaged with GCC.^2^ Given that GCC does not provide a method for re-introducing domain features, we take the same approach as with our ToP models, swapping the projection heads for nodes and edges (GCC+Feat). We also include GCC with positional encodings as features, as in the original work (GCC) (Fig. 3). Alongside the pre-trained GCC models we include results for an equivalent but un-pre-trained model. Where applicable we use the same hyper-parameters as for our fine-tuning of ToP models. In practise this is a batch size of 512 and a learning rate of 0.001.

We find that AD-GCL performs most strongly among the specialist GNN models, MolXPT strongest among the specialist LLMs, Galactica among the generalist LLMs, and ToP-All among the generalist GNNs. Among specialists MolXPT performs most strongly. Among generalists Galactica and our ToP models perform comparably, though both strongly out-perform other models in both classes.

GCC models show consistent negative transfer, both when using their original positional encoding features and when we apply the ToP process for swapping input heads. On the much larger molhiv dataset the smaller (200k parameters vs 2.4M) GCC models reach greater performance within our 50 epoch fine-tuning runs. Performance from GCC models is consistently better when we apply techniques from this work to include specialist features in place of positional encodings using the ToP approach. As the GCC encoders are much smaller than out ToP encoders, we suspect that smaller models trained with ToP would reach comparable performance.

In the Appendix we present few-shot and zero-shot comparisons with LLMs on non-molecular tasks, and find that ToP strongly out-performs all included LLMs on our non-featured graph tasks. Additionally, iterative noising demonstrates that ToP models do indeed carry a bias towards structural information, suggesting that transfer is more effective when tasks are ‘feature-led’, though how to determine which tasks these are a-priori remains an area for future work.

## Discussion

Here we discuss the performance of, and reasonable use cases for, models trained with ToP. We then detail explicitly the assumptions we make and potential risks to validity.

We find that ToP leads to significant positive transfer (Hypothesis 1), and further, feature inclusion downstream does not inhibit this positive transfer. This means, broadly, that ToP can be used without risk of negative transfer. Further, exposure to a larger variety out-of-domain samples can benefit performance. In Table 1, performance increases along the same order as pre-training data diversity (Hypothesis 2). We expect, though we leave this as an area for future research, that a yet greater breadth of domains in the pre-training data would bring greater performance benefits.

### OOD pre-training benefits

One might assume that a non-molecular representation learner would exhibit negative transfer on molecular tasks, and that a molecular learner would exhibit positive transfer, but our ToP-Social and ToP-All show the opposite. Instead the ToP-Social and ToP-All models out-perform the ToP-Chem model on almost all molecular datasets under fine-tuning. This holds true for varied pre-training regimes and model backbones (see Appendix), and shows potential for future work using ToP.

**Fig. 9:**
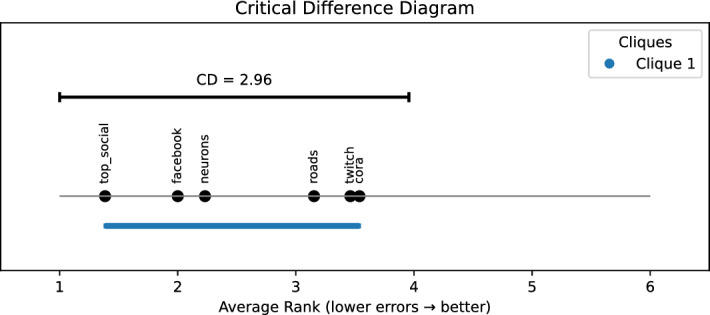
Critical-difference diagram of average ranks for models pre-trained with ToP-Social after ablating each of its five non-chemical datasets (from Table  12). The bold bar denotes the critical difference (CD = 2.96) at $p \le 0.05$. No single dataset’s omission causes a statistically significant shift, suggesting that overall data diversity drives the superior performance of the general social model

This result directly supports Hypothesis 2, and contradicts the assumption that in-domain pre-training is necessary for effective transfer (Liu et al. 2023; Shirzad et al. 2022; Zhang et al. 2023). We propose several explanations for this phenomenon:

- *Structural diversity*. In the absence of features, graph learning relies on patterns in local topology: motifs, connectivity patterns, and multi-node structures. Molecular graphs, though numerous, occupy a constrained region of the structural space. The diversity of the ToP-Social pre-training set, in contrast, exposes the encoder to a wider range of structural motifs.
- *Not driven by single dataset*. Our ablation study (Appendix D, Table 12) excludes the possibility that one particular non-molecular dataset drives this result. We removed each of the five ToP-Social datasets individually and repeated pre-training and evaluation. Nemenyi critical difference analysis (Figure 9, $p \le 0.05$) shows that no single dataset removal produces significant performance change. This demonstrates that overall diversity in the ToP-Social mixture, not similarity between any one dataset and molecules, underpins the superior transfer.

These findings have implications for graph foundation models more broadly. Rather than domain-specific pre-trained models, our results suggest that diverse pre-training across graph domains can provide better transfer than domain-matched data. This requires only that features are excluded during pre-training, then re-introduced during fine-tuning. The same trajectory occurred in vision and language, where foundation models train on broad corpora rather than task-specific data (Chen et al. 2020; Brown 2020). Although theory for this phenomenon in graph representation learning is still developing, similar effects have been observed in the medical image domain, where pre-training on diverse out-of-domain data can outperform or match in-domain pre-training ((Tayebi Arasteh et al. 2024; Juodelyte et al. 2023)).

### Intuitions and comparisons

In comparison to other generalist graph approaches, ToP models perform strongly, including against science-aimed LLM models many orders of magnitude larger in both training data and cost of use. This allows models, with ToP, to be transferred to arbitrary tasks on graph structures, without the costs of LLM-based approaches.

Given these findings the reasonable use-cases for models pre-trained with ToP are broad. The potential benefit is two-fold. Firstly, pre-training and fine-tuning requires less data for the downstream task to be viable. This is particularly valuable in areas of data scarcity, as well as offering presumed decreases in training time for the final model. Secondly, as shown by our results in this work, our pre-trained models offer actual performance increase over simple supervised training. Our assumption is that ToP pre-training introduces a strong bias towards structural information, which in many applications is a positive characteristic, in-particular where it inhibits overfitting to patterns in node or edge features. Guarantees on this point are left for future research.

### Limitations

In the full scope of pre-training and downstream tasks for graph data, this work has some limitations. Firstly we use only static, non-hyper graphs, as in many other graph learning works. We do not experiment with novel graph augmentations, instead employing only node and edge dropping, both random and adversarial. We show that ToP offers performance benefits with random views, and that adversarial views bolster these performance benefits. While adversarial augmentations do improve pre-training, novel augmentations could be developed that include some techniques present in Hassani and Khasahmadi (2020), for example, edge addition alongside edge dropping.

Our method primarily focuses on graph-level tasks, in the same manner as the original AD-GCL work (Suresh et al. 2021). As such, and again as in that original work, we find that performance benefits on node or edge level tasks are much smaller than on graph-level tasks. For node and edge related tasks, future works can optimise ToP through targeted augmentation schemes. Similarly, we employ a limited set of domain compositions in evaluating ToP. A variety of augmentations, paired with a more detailed study of how different compositions of domain data influence representations, could bolster the expressivity of these topology-pre-trained models.

## Conclusion

We present ToP, a method for generalised multi-domain pre-training of graph models. ToP relies on excluding node and edge features during pre-training. Features can then be re-included during fine-tuning on downstream tasks.

With contrastive learning through AD-GCL and GraphCL, we use ToP to present graph models capable of positive transfer across domains and tasks. These are an important step towards graph foundation models with use-cases in the manner of models such as BERT (Devlin et al. 2018). On 75% of downstream tasks from multiple domains we show significant ($p \le 0.01$) positive transfer compared to a non-pre-trained model with the same architecture. This is also true when node and edge features are re-introduced during fine-tuning. Compared to a supervised baseline, where results are not significantly better, they are never significantly worse. Additionally we show that ToP pre-training on a single target domain leads to at-best on-par, and on most tasks significantly worse ($p \le 0.01$), performance compared to pre-training on multiple non-target domains. This clearly demonstrates that the variety provided by out-of-domain pre-training can in this setting hold more value than in-domain pre-training, as in our second hypothesis. We anticipate that future work will focus on several key areas: 1) Improved or tailored graph augmentation strategies. 2) Investigation of domain weighting in dataset compositions. 3) A larger pre-training dataset spanning a wider range of domains. 4) Improved performance on node-level and edge-level tasks. 5) Two-stage pre-training with an in-domain, features-included method. 6) Integration and adaption for graph transformers

## Supplementary Information

Below is the link to the electronic supplementary material.

Supplementary file 1 (pdf 93 KB)

## Figures and Tables

**Fig. 4:**
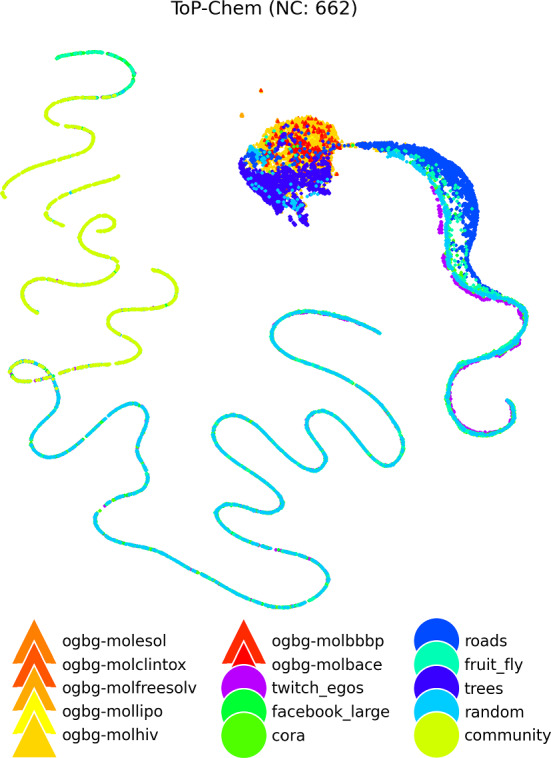
A UMAP embedding of encodings from the ToP-Chem model, pre-trained on only molecules

**Fig. 5:**
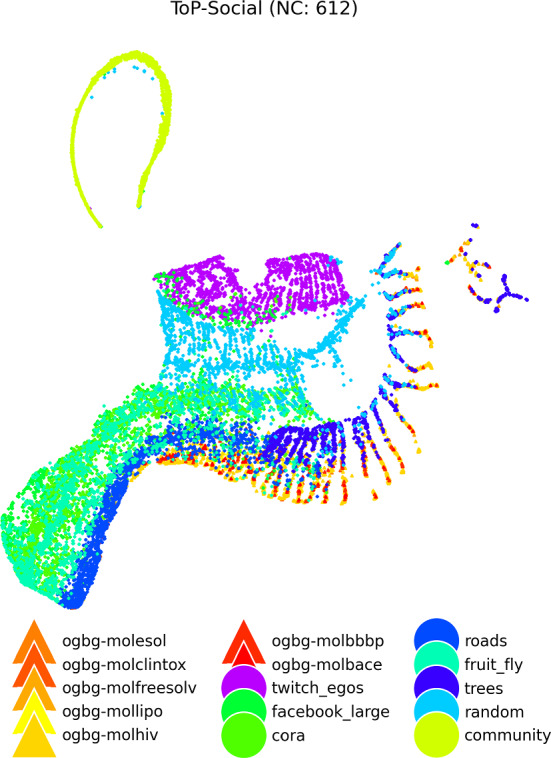
A UMAP embedding of encodings from the ToP-Social model, pre-trained on only non-molecules

**Fig. 6:**
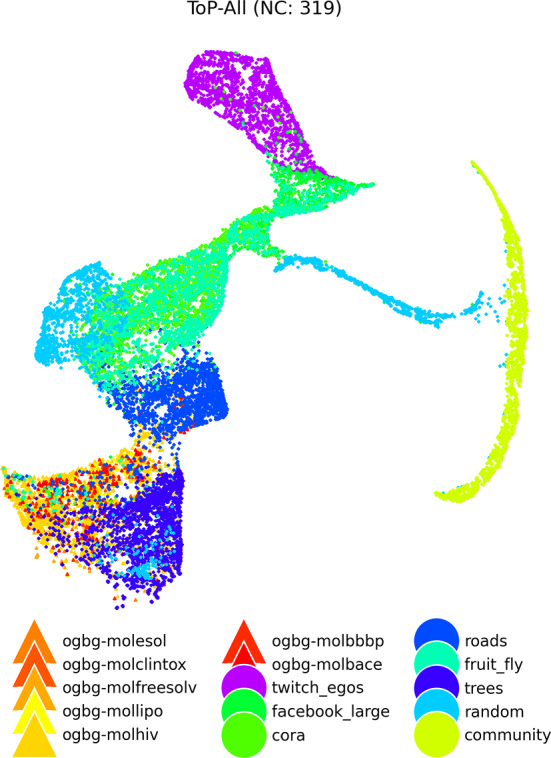
A UMAP embedding of encodings from the ToP-All model, pre-trained on the whole amalgamated dataset

**Fig. 7:**
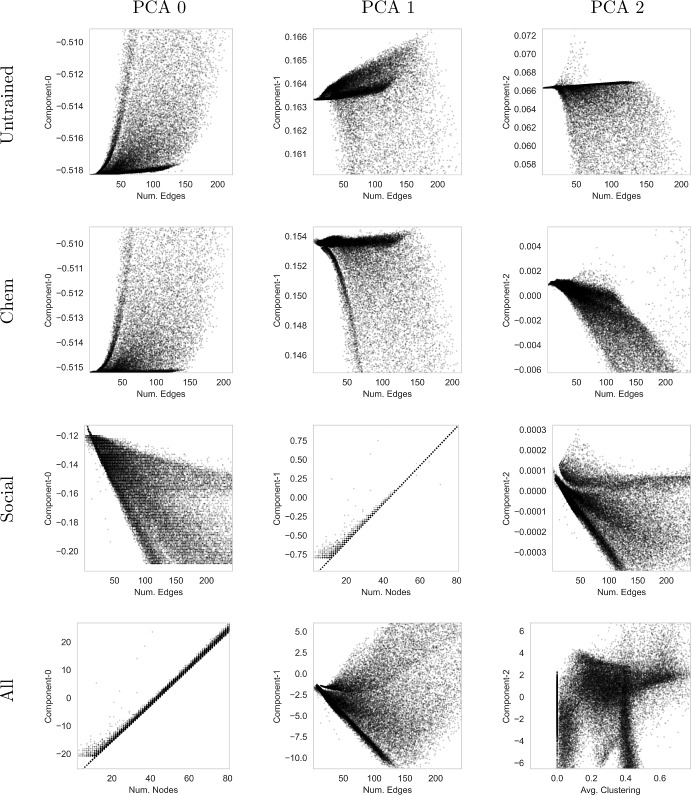
PCA components of the encodings of each model across the whole validation dataset, scattered against the most correlated metrics, as in Table 5

**Fig. 8:**
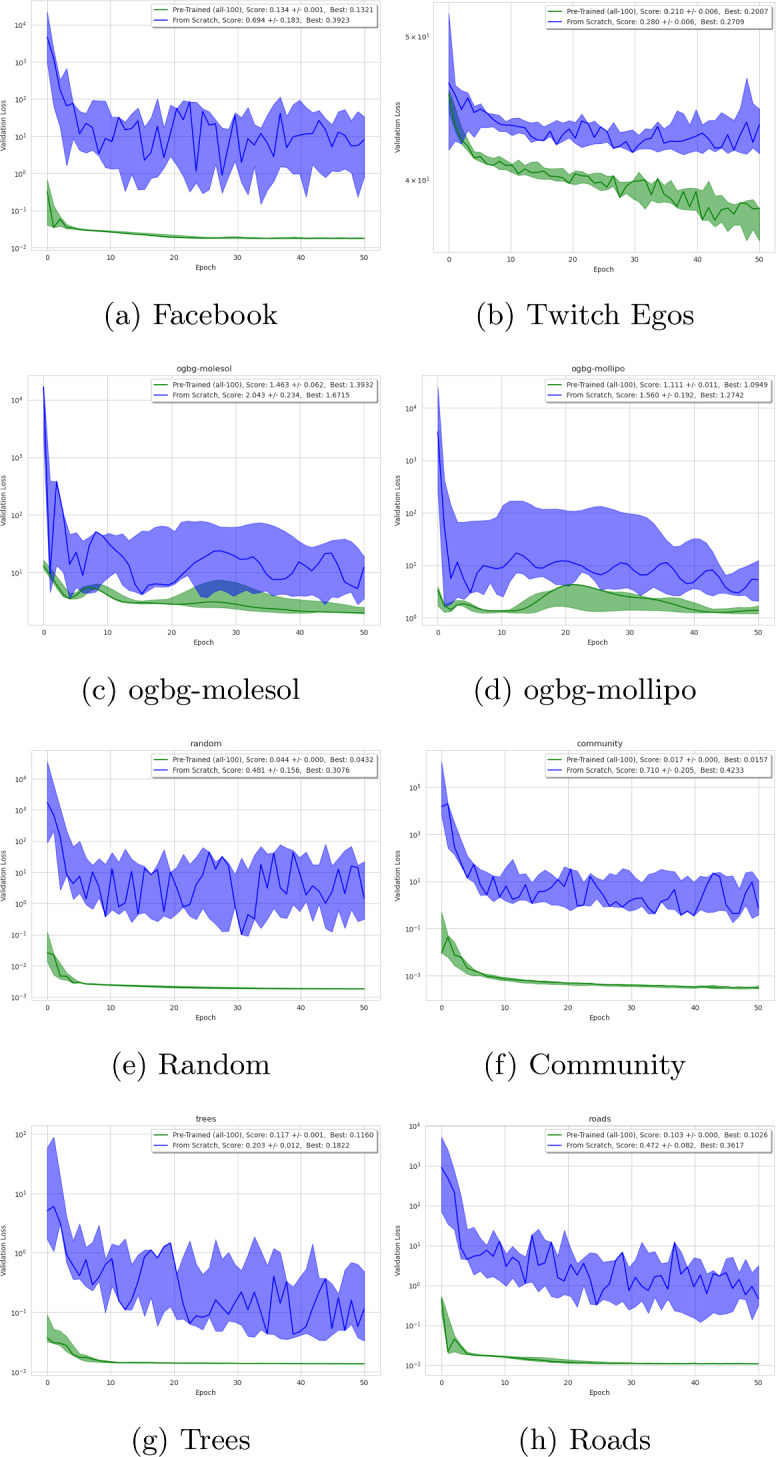
Validation scores for fine-tuning the All model on a subset of our evaluation datasets. The Twitch dataset carries its binary classification task and so we report 1 - AUROC. On other datasets tasks are regression, and we report MSE

**Figure:**
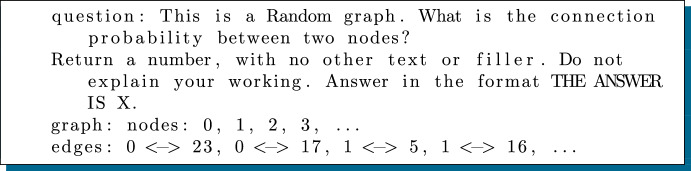
Listing 1 An example prompt for LLMs on the Trees dataset.

**Figure:**
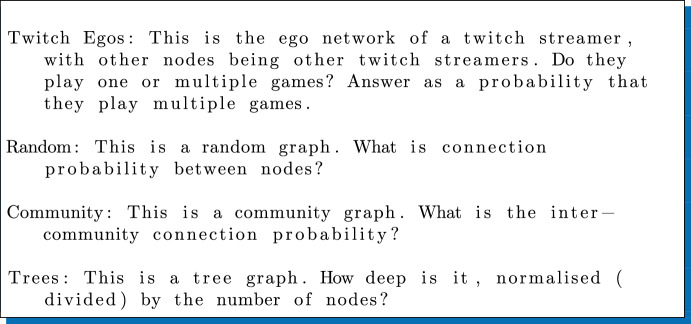
Listing 2 Our task prompts for LLM models.

**Figure:**
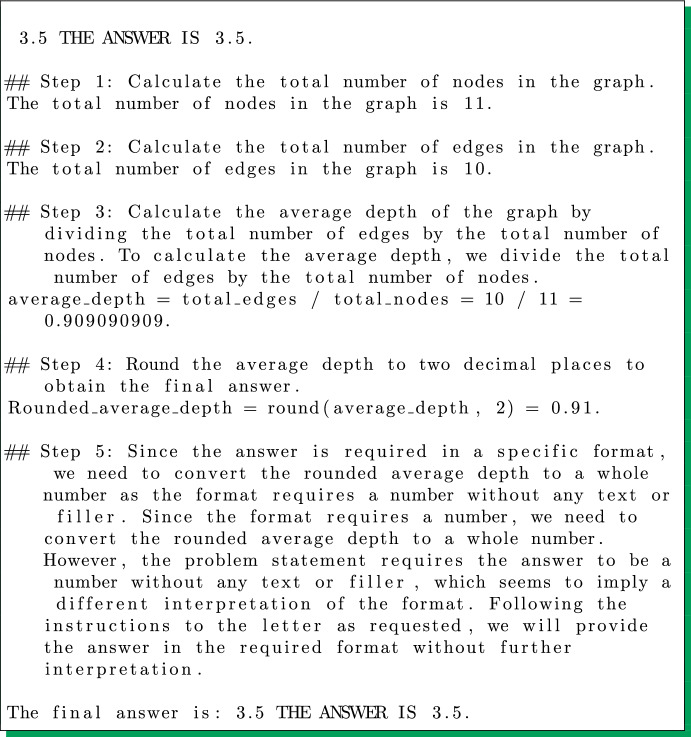
Listing 3 The response to the prompt in Listing 1 from Llama-3.2 (3B). The responsecontains an explanation, despite our prompting specifying not to include any textbeyond the answer.

**Figure:**
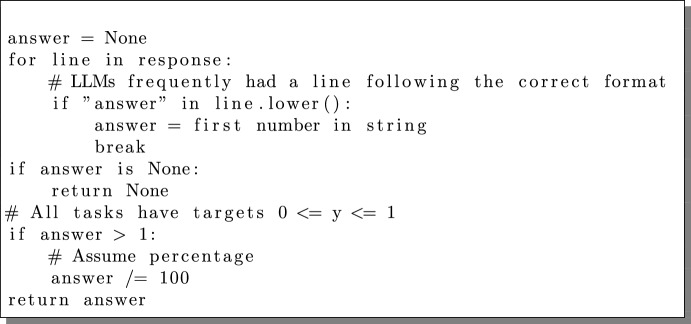
Listing 4 Our process for extracting the predictions of LLMs from generated text.

**Fig. 10:**
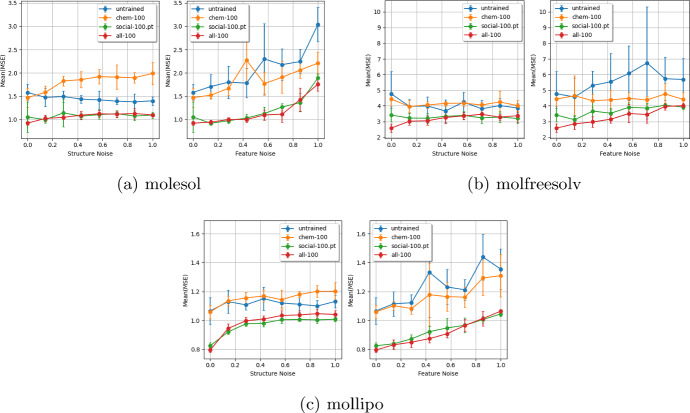
Performance variation for each molecular regression benchmark dataset with increasing noise on structure and features. For each dataset, we benchmark performance for an untrained GIN and our three models pre-trained with ToP (Chem-100, Social-100, All-100)

**Fig. 11:**
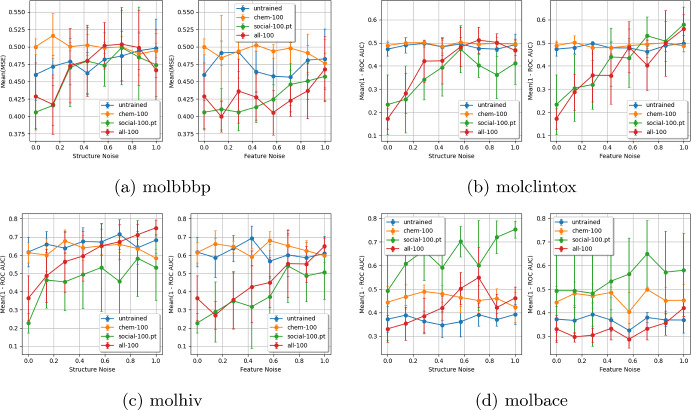
Performance variation for each molecular classification benchmark dataset with increasing noise on structure and features. For each dataset, we benchmark performance for an untrained GIN and our three models pre-trained with ToP (Chem-100, Social-100, All-100)

## Data Availability

No datasets were generated or analysed during the current study.

## Appendix A: Large language models on graphs

Large Language Models (LLMs) are models pre-trained, typically generatively, across massive corpuses of natural language data. Relying on scaling through both dataset size and model size, such models are highly performant on natural language processing. The first foundation models, though trained in the same manner, were smaller and typically required fine-tuning for strong performance on a specific task. Bi-Directional Encoder Representations from Transformers (BERT) is the archetypal example here (Devlin et al. 2018). The most recent LLMs vary in size from a few billion parameters (Aea 2024) to many hundreds of billions (Ray 2023).

LLMs have in very recent research been applied to building multi-domain graph models, often termed Graph Foundation Models (GFMs) in their respective works. Some have argued that LLMs themselves, by default, may be GFMs (Mao et al. 2024). LLMs carry the significant benefit of allowing one-shot or few-shot application on downstream tasks in some circumstances. However, given their size, LLMs can drastically increase the cost and complexity of using graph models.

The simplest approach for applying LLMs on graph data is simply to encode a graph structure in natural language, along with features. We model our description of this approach on Fatemi et al. (2024). Here a graph structure is encoded via some schema, for example the edge (*Callum*, *Zack*) might be encoded *‘Callum and Zack are connected’* in a social network. Fatemi et al. (2024) find that the choice of encoding schema, for example *‘Oli is friends with Michael’* vs *‘Clem is connected to Tom’*, has a significant impact on performance, and that in-general this approach is limited.

Alternatively the performant capacity of LLMs as encoders has been used to unify the graph feature space across domains. By describing the features of a node or edge in natural language, the LLMs encodings of that new textual feature can be shared between domain feature sets (Shang et al. 2024; Zhang et al. 2024; Xu et al. 2025; Liu et al. 2023a). This assumes and requires that:

1. Original features are easily encoded in text
2. Natural language versions of features are meaningful
3. LLM encodings are in fact a coherent embedding space

For natural-language features, the approach is trivial, and numeric features are converted to text via a schema. An example for molecular graphs would be *‘An atom with X protons, Y covalent bonds, which (is/is not) part of a ring structure’*. These textual features are then passed to an LLM to form numeric encodings as features. This has the significant advantage of allowing one-shot or few-shot downstream use, assuming that downstream text features are close to in-domain. The approach has yet to be applied to graphs with rich feature sets. We assume this because it is not clear how previous approaches would encode non-intuitive graph features textually, such as continuous ones. Some of these initial works use multiple domains where features are already text, and do not attempt to move other feature sets into textual features.

Another approach has emerged in LLMs for chemistry, where there are expressive sequential descriptions of molecular graphs commonly used in natural language (Liu et al. 2023b; Zhao et al. 2025; Cao et al. 2025). Here LLM approaches use sequential SMILES encodings of molecules, for example O=C=O from CO$_2$, to represent molecules at some point of their pipeline. SMILES strings, as a natural language expression of a graph structure, are a useful tool for LLM approaches. Most other graph structures, for example social networks, are much harder to express in natural language. A core assumption of these models, with strong evidence for validity through experimentation, is that forming datasets of text surrounding these SMILES encodings using chemical literature allows learning about chemical dynamics to occur. Where these models do not include explicit usage of graph structures, for example InstructMol (Cao et al. 2025) and ChemDFM (Zhao et al. 2025), they require far larger models and datasets for the same performance.

**Table 4:** Statistics for each of our datasets

	Num. Graphs	Number of Nodes	Number of Edges	Diameter	Avg. Clustering	Task
*Training Only*						
molpcba	250000	25.7 ± 6.28	27.7 ± 7	13.5 ± 3.29	0.00128 ± 0.0115	–
Cora	50000	59.3 ± 20.7	119± 56.6	10.1 ± 4.68	0.322 ± 0.0845	–
Fly Brain	50000	59.5 ± 20.8	146± 99.6	8.8 ± 3.49	0.237 ± 0.0875	–
*Training and Evaluation*						
Twitch	50000	29.6 ± 11.1	86.6 ± 70.7	2± 0	0.549 ± 0.149	Class. Games
Facebook	50000	59.5 ± 20.7	206 ± 170	10.2 ± 6.39	0.429 ± 0.133	Avg. Clust
Roads	50000	59.5 ± 20.8	73.9 ± 28.2	16.4 ± 5.92	0.0559 ± 0.0426	Diameter / *V*
*Evaluation Only*						
molesol	1015	12.7 ± 6.64	12.9 ± 7.62	6.81 ± 3.30	0.000353 ± 0.00417	Solubility
molfreesolv	577	8.46 ± 3.97	8.02 ± 4.50	5.02 ± 2.09	0.0 ± 0.0	Solvation
mollipo	3780	27.0 ± 7.44	29.4 ± 8.22	13.8 ± 4.03	0.00366 ± 0.017	Lipophilicity
molclintox	1329	26.3 ± 15.8	28.0 ± 17.1	12.4 ± 6.09	0.00259 ± 0.0191	Class. Tox
molbbbp	1835	23.5 ± 9.89	25.4 ± 11.0	11.2 ± 4.03	0.00284 ± 0.0278	Class. Pen
molbace	1361	34.0 ± 7.89	36.8 ± 8.13	15.2 ± 3.14	0.00672 ± 0.0205	Class. Bioact
molhiv	37014	25.3 ± 12.0	27.3 ± 13.1	12.0 ± 5.15	0.00158 ± 0.0156	Class. Inhib
*Evaluation Only, Unseen Domain*						
Trees	5000	19.6 ± 6.96	18.6 ± 6.96	10.1 ± 3.11	0± 0	Depth
Random	5000	29.3 ± 10.7	111± 82.1	3.88 ± 1.16	0.227 ± 0.0998	Density
Community	5000	48± 0	323± 26.2	3± 0.0346	0.408 ± 0.0256	Inter-Comm. Density

If used for training, we denote these statistics averaged across the training data. Otherwise we report statistics for their validation (downstream fine-tuning) datasets. All tasks are single-target

**Table 5:** On the combined encodings of the validation sets we compute $R^2$ correlation coefficients between the first five principal components (PCA) and common graph-level metrics

	Variance Ratio	Metric Correlations					
		Most	$R^2$	Second	$R^2$	Third	$R^2$
*Untrained*							
PCA 0	$9.96\times 10^{-01}$	Edges	0.219	Transitivity	0.072	Density	0.063
PCA 1	$3.52\times 10^{-03}$	Edges	**−0.555**	Transitivity	−0.276	Density	−0.243
PCA 2	$1.13\times 10^{-04}$	Edges	**0**.**39**	Transitivity	0.224	Density	0.21
*Chem*							
PCA 0	$9.97\times 10^{-01}$	Edges	0.223	Transitivity	0.074	Density	0.065
PCA 1	$2.60\times 10^{-03}$	Edges	**−0.514**	Transitivity	−0.253	Density	−0.225
PCA 2	$1.14\times 10^{-05}$	Edges	0.132	Nodes	0.051	Transitivity	0.047
*Social*							
PCA 0	$9.99\times 10^{-01}$	Edges	0.127	Transitivity	0.032	Density	0.032
PCA 1	$9.92\times 10^{-04}$	Nodes	**1.00**	Edges	**0**.**528**	Density	**−0.393**
PCA 2	$4.79\times 10^{-08}$	Edges	0.133	Density	0.07	Transitivity	0.054
*All*							
PCA 0	$7.04\times 10^{-01}$	Nodes	**0.999**	Edges	**0**.**534**	Density	**−0.388**
PCA 1	$1.94\times 10^{-01}$	Edges	**0.805**	Density	**0.737**	Diameter	**−0.554**
PCA 2	$4.23\times 10^{-02}$	Clustering	0.309	Transitivity	0.248	Edges	0.235
PCA 3	$2.43\times 10^{-02}$	Diameter	**0**.**5**	Clustering	**−0.416**	Transitivity	−0.243
PCA 4	$1.82\times 10^{-02}$	Density	−0.132	Transitivity	−0.108	Diameter	−0.088

High correlations, or the lack thereof, should give some indication of how embedding components represent different graph characteristics. Here we present the most correlated metrics and components for each pre-trained model. Underlined text indicates a strong correlation $|R^2| \ge 0.66$, and bold text indicates moderate correlation $0.33 \le |R^2| \le 0.66$

**Table 6:** Scores for linear models applied to the encodings of each validation dataset, with and without node labels

	Untrained	Chem	Social	All	Mag.
molfreesolv	4.21 ± 4.13	3130 ± 5990	2.00 ± 0.869	**1**.**01** ±**1**.**02**	$10^3$
molesol	48.3 ± 90.2	817 ± 1150	48.1 ± 62.9	**28**.**1**±**38**.**1**	$10^0$
mollipo	29.4 ± 17.9	14.9 ± 2.0	**4**.**45** ±**0**.**62**	5.33 ± 0.56	$10^0$
molclintox	0.487 ± 0.005	0.440 ± 0.007	**0**.**500**±**0**.**000**	**0**.**500**±**0**.**000**	$10^0$
Facebook	10.6 ± 1.7	10.7 ± 20	7.73 ± 1.87	**4**.**44** ±**5**	$10^{-3}$
Twitch Egos	0.638 ± 0.012	0.610 ± 0.072	0.679 ± 0.002	**0**.**694** ±**0**.**002**	$10^0$
Roads	263 ± 0.5	256 ± 0.0	298 ± 0.3	**2**.**85** ±**5**	$10^{-5}$
Trees	7.66 ± 0.16	2.85 ± 0.0	2.98 ± 0.03	**2**.**56** ±**0**.**05**	$10^{-3}$
Community	4.36 ± 0.05	**4**.**25** ±**0**.**00**	12.0 ± 2	4.46 ± 0.07	$10^{-5}$
Random	6.46 ± 0.7	3.27 ± 0.0	**1**.**20** ±**0**.**2**	3.38 ± 0.0	$10^{-4}$

For the classification datasets (ogbg-molclintox and Twitch Egos) we report AUROC, other datasets report RMSE. Some datasets, where results are very similar for each model, are excluded. Bold text indicates a superior result. For clarity the results for a given dataset are scaled by a constant factor

**Table 7:** Scores for fine-tuning models trained with random node dropping on each validation dataset, with (Model-labels) and without (Model) node labels

	GIN	Chem	Social	All
molfreesolv	4.978 ± 0.479	$\underline{\boldsymbol{3.838} \pm \boldsymbol{0.120}}$	**4**.**041** ±**0**.**116**	**4**.**157** ±**0**.**138**
molesol	1.784 ± 0.094	1.897 ± 0.075	**1**.**520** ± **0**.**066**	$\underline{\boldsymbol{1.357} \pm \boldsymbol{0.037}}$
mollipo	1.143 ± 0.031	**1**.**123** ± **0**.**105**	**1**.**015** ±**0**.**013**	$\underline{\boldsymbol{0.983} \pm \boldsymbol{0.015}}$
molclintox	$\underline{0.530 \pm 0.042}$	0.469 ± 0.030	0.431 ± 0.002	$\underline{\boldsymbol{0.530} \pm \boldsymbol{0.086}}$
molbbbp	$\underline{0.565 \pm 0.043}$	0.497 ± 0.027	0.548 ± 0.037	$\underline{\boldsymbol{0.567} \pm \boldsymbol{0.032}}$
molbace	0.677 ± 0.053	$\underline{\boldsymbol{0.740} \pm \boldsymbol{0.026}}$	**0**.**686** ±**0**.**055**	**0**.**679** ±**0**.**067**
molhiv	0.355 ± 0.059	0.283 ± 0.001	**0**.**635** ±**0**.**046**	$\underline{\boldsymbol{0.644} \pm \boldsymbol{0.039}}$
Facebook	0.311 ± 0.065	**0**.**261** ± **0**.**101**	**0**.**159** ±**0**.**015**	$\underline{\boldsymbol{0.145} \pm \boldsymbol{0.012}}$
Twitch Egos	0.72 ± 0.006	0.460 ± 0.041	$\underline{\boldsymbol{0.744} \pm \boldsymbol{0.006}}$	$\underline{\boldsymbol{0.743} \pm \boldsymbol{0.003}}$
Roads	0.46 ± 0.073	**0**.**228** ±**0**.**118**	**0**.**150** ± **0**.**015**	$\underline{\boldsymbol{0.132} \pm \boldsymbol{0.018}}$
Trees	0.203 ± 0.012	0.415 ± 0.250	**0**.135 ± 0.015	$\underline{\boldsymbol{0.125} \pm \boldsymbol{0.009}}$
Community	0.743 ± 0.106	**0**.**084** ± **0**.**083**	**0**.**080** ± **0**.**018**	$\underline{\boldsymbol{0.074} \pm \boldsymbol{0.021}}$
Random	0.529 ± 0.228	**0**.**123** ± **0**.**075**	**0**.**076** ± **0**.**010**	$\underline{\boldsymbol{0.058} \pm \boldsymbol{0.005}}$
	**GIN**-Labels	**Chem**-Labels	**Social**-Labels	**All**-Labels
molfreesolv	4.854 ± 0.513	$\underline{\boldsymbol{3.819} \pm \boldsymbol{0.136}}$	**4**.**113** ±**0**.**070**	**3**.**984** ±**0**.**162**
molesol	1.707 ± 0.073	1.809 ± 0.139	**1**.**480** ±**0**.**067**	$\underline{\boldsymbol{1.378} \pm \boldsymbol{0.031}}$
mollipo	1.143 ± 0.031	1.182 ± 0.128	$\underline{\boldsymbol{0.986} \pm \boldsymbol{0.017}}$	**0**.**997** ±**0**.**011**
molclintox	$\underline{0.497 \pm 0.038}$	0.474 ± 0.036	0.430 ± 0.002	0.474 ± 0.056
molbbbp	$\underline{0.561 \pm 0.034}$	0.491 ± 0.032	0.543 ± 0.016	$\underline{\boldsymbol{0.565} \pm \boldsymbol{0.036}}$
molbace	0.698 ± 0.050	$\underline{\boldsymbol{0.751} \pm \boldsymbol{0.024}}$	0.677 ± 0.030	0.675 ± 0.088
molhiv	0.374 ± 0.052	0.286 ± 0.008	$\underline{\boldsymbol{0.615} \pm \boldsymbol{0.047}}$	$\underline{\boldsymbol{0.628} \pm \boldsymbol{0.044}}$
Facebook	0.749 ± 0.250	**0**.**354** ± **0**.**166**	$\underline{\boldsymbol{0.151} \pm \boldsymbol{0.016}}$	**0**.**156** ±**0**.**022**
**Superior Result**	19.0%	19.0%	19.0%	**61.9%**
**Beats Baseline**	-	47.6%	76.2%	**90.5%**

For the classification datasets (ogbg-molclintox and Twitch Egos) we report AUROC, other datasets report RMSE. Some datasets, where results are very similar for each model, are excluded. Bold text indicates a result beats the unsupervised (GIN) baseline. Underlined text indicates a superior result, down to three significant figures

**Table 8:** Scores for fine-tuning models trained with random edge dropping on each validation dataset, with (Model-labels) and without (Model) node labels

	GIN	Chem	Social	All
molfreesolv	4.978 ± 0.479	134.800± 225.200	$\underline{\boldsymbol{4.102} \pm \boldsymbol{0.049}}$	**4**.**197** ± **0**.**095**
molesol	1.784 ± 0.094	14.050 ± 23.760	$\underline{\boldsymbol{1.312} \pm \boldsymbol{0.094}}$	**1**.**571** ± **0**.**059**
mollipo	1.129 ± 0.040	**1**.**034** ± **0**.**029**	$\underline{\boldsymbol{0.989} \pm \boldsymbol{0.011}}$	**1**.**068** ± **0**.**026**
molclintox	0.530 ± 0.042	0.449 ± 0.013	0.426 ± 0.006	0.461 ± 0.029
molbbbp	0.565 ± 0.043	**0**.**570** ± **0**.**040**	$\underline{\boldsymbol{0.602} \pm \boldsymbol{0.025}}$	$\underline{\boldsymbol{0.603} \pm \boldsymbol{0.029}}$
molbace	0.677 ± 0.053	**0**.**768** ± **0**.**006**	$\underline{\boldsymbol{0.783} \pm \boldsymbol{0.045}}$	**0**.**756** ± **0**.**030**
molhiv	0.355 ± 0.059	**0**.**576** ± **0**.**152**	$\underline{\boldsymbol{0.647} \pm \boldsymbol{0.066}}$	**0**.**501** ± **0**.**135**
Facebook	0.311 ± 0.065	3.70 ± 6.95	$\underline{\boldsymbol{0.180} \pm \boldsymbol{0.019}}$	**0**.237 ± 0.063
Twitch Egos	0.72 ± 0.006	0.717 ± 0.005	$\underline{\boldsymbol{0.753} \pm \boldsymbol{0.006}}$	**0**.739 ± 0.007
Roads	0.46 ± 0.073	$\underline{\boldsymbol{0.156} \pm \boldsymbol{0.007}}$	**0**.**167** ± **0**.**039**	** 0.252 ** ± **0.029**
Trees	0.203 ± 0.012	$\underline{\boldsymbol{0.143} \pm \boldsymbol{0.009}}$	**0**.**161** ± **0**.**019**	** 0.187 ** ± ** 0.020**
Community	0.743 ± 0.106	$\underline{\boldsymbol{0.014} \pm \boldsymbol{0.000}}$	**0**.**146** ± **0**.**024**	** 0.314 ** ± **0.069**
Random	0.529 ± 0.228	$\underline{\boldsymbol{0.055} \pm \boldsymbol{0.012}}$	**0**.**107** ± **0**.**019**	** 0.289 ** ± **0.098**
	**GIN**-Labels	**Chem**-Labels	**Social**-Labels	**All**-Labels
molfreesolv	4.854 ± 0.513	103.400±232.200	$\underline{\boldsymbol{4.106} \pm \boldsymbol{0.021}}$	**4**.**163** ± **0**.**069**
molesol	1.707 ± 0.073	8.006 ± 7.850	$\underline{\boldsymbol{1.358} \pm \boldsymbol{0.028}}$	**1**.**597** ± **0**.**069**
mollipo	1.143 ± 0.031	**1**.**043** ± **0**.**026**	$\underline{\boldsymbol{0.995} \pm \boldsymbol{0.014}}$	**1**.**063** ± **0**.**038**
molclintox	0.497 ± 0.038	0.446 ± 0.020	0.422 ± 0.009	0.475 ± 0.031
molbbbp	0.561 ± 0.034	0.535 ± 0.079	$\underline{\boldsymbol{0.612} \pm \boldsymbol{0.015}}$	**0**.**589** ± **0**.**031**
molbace	0.698 ± 0.050	**0**.**763** ± **0**.**018**	$\underline{\boldsymbol{0.776} \pm \boldsymbol{0.032}}$	**0**.**744** ± **0**.**034**
molhiv	0.374 ± 0.052	**0**.**632** ± **0**.**087**	$\underline{\boldsymbol{0.652} \pm \boldsymbol{0.100}}$	**0**.**479** ± **0**.**102**
Facebook	0.749 ± 0.250	2.80 ± 4.14	$\underline{\boldsymbol{0.179} \pm \boldsymbol{0.020}}$	**0**.**263** ± **0**.**067**
**Superior Result**	9.5%	19.0%	**71.4%**	4.8%
**Beats Baseline**	-	52.4%	**90.5%**	**90.5%**

For the classification datasets (ogbg-molclintox and Twitch Egos) we report AUROC, other datasets report RMSE. Some datasets, where results are very similar for each model, are excluded. Bold text indicates a result beats the unsupervised (GIN) baseline. Underlined text indicates a superior result, down to three significant figures

**Table 9:** Results for fine-tuning for three different layer architectures (Graph Convolution Network, Graph Attention Network, Graph Isomorphism Network) and pre-training data split on molecular benchmark datasets with node and edge features included

Layer	Data	freesolv	esol	lipo	bbbp	clintox	bace	hiv
GCN	None	2.46 2.92 0.309	1.32 1.507 0.095	1.02 1.14 0.066	0.303 0.335 0.025	0.0783 0.126 0.036	0.177 0.233 0.036	0.466 0.589 0.074
	Chem	2.38 3.01 0.456	0.881 0.977 0.072	0.745 0.782 0.016	0.329 0.375 0.029	0.150 0.532 0.135	0.239 0.292 0.038	0.255 0.371 0.064
	Social	1.87 2.62 0.363	0.896 0.965 0.055	0.739 0.765 0.027	0.318 0.352 0.02	0.0879 0.305 0.201	0.195 0.28 0.067	0.249 0.345 0.119
	All	2.29 2.86 0.38	1.00 1.10 0.094	0.779 0.827 0.042	0.327 0.387 0.029	0.0663 0.468 0.134	0.200 0.27 0.048	0.245 0.352 0.072
GAT	None	2.55 3.02 0.292	1.15 1.33 0.116	0.897 1.11 0.097	0.324 0.352 0.022	0.0471 0.157 0.058	0.156 0.243 0.039	0.536 0.63 0.063
	Chem	3.87 4.02 0.113	1.40 1.69 0.192	0.911 1.03 0.05	0.329 0.433 0.058	0.5 0.5 0.002	0.238 0.378 0.085	0.286 0.514 0.166
	Social	3.45 3.92 0.271	1.07 1.49 0.262	0.979 1.06 0.093	0.322 0.391 0.044	0.5 0.5 0	0.268 0.371 0.08	0.308 0.397 0.082
	All	3.34 4.16 0.549	1.08 1.93 0.636	0.936 1.03 0.064	0.369 0.416 0.041	0.489 0.498 0.003	0.270 0.339 0.061	0.283 0.509 0.15
GIN	None	3.22 4 0.434	1.48 1.72 0.245	1.02 1.09 0.053	0.357 0.399 0.03	0.112 0.351 0.124	0.210 0.247 0.025	0.611 0.679 0.032
	Chem	3.66 4.16 0.288	1.25 1.62 0.306	1.05 1.09 0.056	0.347 0.391 0.027	0.444 0.485 0.025	0.243 0.305 0.073	0.533 0.626 0.066
	Social	2.98 3.61 0.428	0.856 1.03 0.165	0.759 0.798 0.023	0.334 0.374 0.026	0.106 0.138 0.023	0.232 0.304 0.05	0.278 0.383 0.106
	All	2.00 3.01 0.594	0.807 0.897 0.042	0.759 0.788 0.016	0.290 0.335 0.021	0.0543 0.164 0.054	0.203 0.276 0.048	0.272 0.434 0.131

On regression datasets (freesolv, esol, lipo) we report MSE, and on classification datasets (bbbp, clintox, bace, hiv) we report 1 - AUROC

Each cell shows Best/Mean/± Dev. values from top to bottom

## Appendix B: Node and edge transfer

As in graph-level transfer we replace the original input head with a three-layer MLP for both node classification and edge prediction. For node classification, we use a single-layer MLP as an output head. For edge prediction, the embeddings from the encoder are concatenated for the relevant nodes, then passed to a single-layer MLP.

For edge prediction we sample the two-hop ego network of each node from the original graph. For node prediction we take the three-hop ego network, but limit the scale to five neighbours per node. As larger graphs we employ the Facebook and Cora graphs, as well as the Citeseer and PubMed graphs from the same paper. For edge prediction we also include the Amazon Computers and Photo graphs from Shchur et al. (2018). For node classification we also employ the GitHub and LastFM Asia graphs from Rozemberczki et al. (2021). For edge prediction we take 5% splits for training and testing edges. For node classification we take a 20% split. The results of this transfer can be found in Table 10.

**Table 10:** Scores for fine-tuning the complete model with node and edge features for node classification and edge prediction. For both node classification and edge prediction we report accuracy

Edge Pred.	GIN	ToP-All (CL)	Chem	Social	All
Amzn. Comp	0.85 ± 0.06	0.82 ± 0.08	0.84 ± 0.04	**0**.**86** ± **0**.**05**	$\underline{\boldsymbol{0.86} \pm \boldsymbol{0.03}}$
Amzn. Photo	0.86 ± 0.04	0.86 ± 0.05	**0**.**89** ± **0**.**03**	**0**.**88** ± **0**.**05**	$\underline{\boldsymbol{0.91} \pm \boldsymbol{0.03}}$
Citeseer	$\underline{0.81 \pm 0.01}$	0.78 ± 0.02	0.75 ± 0.02	0.75 ± 0.01	0.80 ± 0.01
PubMed	0.97 ± 0.01	$\underline{\boldsymbol{0.98} \pm \boldsymbol{0.01}}$	0.96 ± 0.02	$\underline{\boldsymbol{0.98} \pm \boldsymbol{0.01}}$	**0**.**97** ± **0**.**01**

Node Class.	GIN	–	Chem	Social	All
Citeseer	$\underline{0.75 \pm 0.01}$	0.71 ± 0.05	0.72 ± 0.04	0.72 ± 0.01	0.74 ± 0.01
GitHub	0.89 ± 0.00	0.89 ± 0.02	$\underline{\boldsymbol{0.89} \pm \boldsymbol{0.00}}$	0.88 ± 0.02	0.89 ± 0.00
LastFM	0.79 ± 0.00	0.76 ± 0.02	0.78 ± 0.01	0.79 ± 0.01	$\underline{\boldsymbol{0.79} \pm \boldsymbol{0.00}}$
PubMed	0.47 ± 0.00	**0**.**47** ± **0**.**00**	**0**.**47** ± **0**.**00**	**0**.**47** ± **0**.**00**	$\underline{\boldsymbol{0.47} \pm \boldsymbol{0.00}}$

Bold formatting indicates that a result is on-average superior to an untrained GIN baseline; an underlined result indicates that a model on-average performs above all other models presented

## Appendix C: LLMs on non-molecular tasks

We apply two recent LLMs, ChatGPT-4o-mini (Openai et al. 2023) and Llama−3.2-Instruct (3B) (Aea 2024), across our validation-only datasets (random, community, trees), as well as the Twitch Egos dataset. We use these smaller versions from their respecitve LLM families to reduce computational cost, which is still drastically higher than our GNN-only approach. From here we denote Llama−3.2-Instruct (3B) as Llama-3. These tasks are chosen as they do not rely on features, meaning that learning and performance directly follows the use of structural information. We also include GCC with positional encodings as features, as in the original work.

### LLM prompting

Using LLMs for arbitrary graph structures requires defining a schema for natural language representations of graphs. We opt for the ‘incident’ encoding style from Fatemi et al. (2024). In this format a graph is encoded in the style *nodes*: (1, 2, 3, ...) *edges:* $(1\leftrightarrow 2, 2\leftrightarrow 3,...)$. We define prompts for each dataset, aimed to both provide contextual information and make tasks clear, which can be found below. We include further prompting text in an attempt to ensure uniformity across responses: *‘Return a number, with no other text or filler. Do not explain your working. Answer in the format THE ANSWER IS X.’* The prompt structure is then {task description + extra prompt information + graph information}. Below we show an example prompt and response from LLaMa-3, as well as our method for extracting numerical answers from responses (Fig. 4 and 5).

**Table 11:** Our results for the ToP-All and ToP-Social models presented alongside LLMs and GCC applied to structure-only datasets

	Twitch Egos	Random	Community	Trees
GPT-4o-mini (8B)	0.50	0.30	0.37	0.28
LLaMa−3.2 (3B)	0.46	0.12	0.10	0.42
GCC (untrained)	0.66 ± 0.01	**0**.**03** ± **0**.**00**	**0**.**02** ± **0**.**01**	**0**.**12** ± **0**.**01**
GCC (200k)	0.66 ± 0.01	**0**.**03** ± **0**.**00**	**0**.**02** ± **0**.**00**	**0**.**12** ± **0**.**00**
ToP-**Social** (2.4M)	0.74 ± 0.01	0.04 ± 0.00	**0**.**02** ± **0**.**00**	**0**.**12** ± **0**.**00**
ToP-**All**	**0**.**79** ± **0**.**01**	0.04 ± 0.00	**0**.**02** ± **0**.**00**	**0**.**12** ± **0**.**00**

Bold formatting indicates a model performs on-average above the other models presented.

Random, Community and Trees are unseen data for all models. Twitch Egos is AUC-ROC, other datasets are RMSE. Due to cost constraints we do not produce error margins for LLM models

*Findings* Our results are presented in Table 11.^3^,^4^ LLMs, across all tasks, perform worse than ToP or GCC models. This is despite three of our datasets consisting of basic counting tasks, meaning that they are possible to perform in natural language. Larger LLMs might perform better, but given that these models are already many orders of magnitude larger than ToP or GCC models, we view the LLMs used here as fair benchmarks. On the three synthetic datasets (Random, Community, Trees), with their simple structure-based tasks, ToP models and GCC reach essentially the same performance. On the real-world Twitch Egos dataset ToP models strongly out-perform the LLMs and GCC, and our ablation in Sect. 10.1 demonstrates this is not an artefact of the pre-training data for ToP models (Figs. 6, 7, 8 and Tables 4, 5, 6, 7, 8, 9).

## Appendix D: Ablation & structural bias

Having demonstrated the efficacy of ToP, we present two more experiments, designed to give some insight into why and how ToP pre-training is effective. The first ensures that our results supporting Hypothesis 2, that out-of-domain data can be more useful than in-domain, are not the result of artefacting. The second investigates the extent to which ToP, during downstream transfer, relies on feature information in comparison to structural information. Additional results for edge and node level tasks can be found in the Appendix, as well as transfer with a linear models, ToP pre-trained with random GraphCL-like augmentations, and ToP with varied GNN backbones.

Our ablation study demonstrates that our results are not a product of arte-facting from pre-training data, as excluding individual datasets does not then produce downstream negative transfer at $p=0.05$.

While the results we present are statistically significant, there remains the possibility of artefacting. In particular it is feasible that a particular domain in the ToP-Social pre-training data lead to positive transfer, not the overall diversity of data. We exclude one of the five pre-training datasets at a time, pre-train an encoder with ToP, then fine-tune ten times across our validation datasets, under the same transfer settings as our main results. We do not increase the size of the Social pre-training data. We find that no individual ablation produces a statistically significant performance gap ($p=0.05$), emphasising that it is the overall diversity of the social pre-training data, not any particular domain, that underpins the consistently superior performance of the ToP-Social model. The exact performances and respective Nemenyi critical difference diagram are presented in the Appendix.

Having established both that ToP models learn useful structural information *I*(*y*; *E*), and further that these models can then re-integrate feature information *I*(*y*; *X*, *E*), this section determines to what degree ToP models rely on information from either source. We use noise functions, destroying information in either structure or features, and evaluating performance as this information is degraded. A further discussion for this method, along with visualisations, is provided in the Appendix.

We find that the untrained GIN model and ToP-Chem have a smaller response to structural noise than ToP-Social and ToP-All, and have a larger response to feature noise. This indicates that ToP, over varied pre-training data, introduces a bias towards using structural information. Further, datasets on which ToP provides smaller performance benefits show minimal performance change from all models under structural noise, indicating that these tasks rely more heavily on feature information. In other terms, they can be described as ‘feature-led’, in contrast to tasks where structural information is more useful (eg. molclintox). This means that ToP is a more useful pre-training method where tasks are ‘structure-led’ instead of ‘feature-led’. How to determine which tasks these are remains an area for future research.

### Ablation study critical difference

**Table 12:** Scores for fine-tuning models pre-trained on non-molecules, each with one non-molecular pre-training dataset excluded

	Social (Name indicates excluded dataset)				
	Twitch	Neurons	Cora	Facebook	Roads
Facebook	**0**.**29** ± **0**.**02**	**0**.**14** ± **0**.**00**	**0**.**84** ± **0**.**01**	**0**.**14** ± **0**.**01**	**0**.**22** ± **0**.**06**
Twitch Egos	**0**.**73** ± **0**.**03**	**0**.**74** ± **0**.**01**	**0**.**73** ± **0**.**01**	**0**.**74** ± **0**.**01**	**0**.**77** ± **0**.**01**
Roads	**0**.**19** ± **0**.**02**	**0**.**11** ± **0**.**00**	**0**.**12** ± **0**.**01**	**0**.**11** ± **0**.**00**	**0**.**18** ± **0**.**01**
Trees	**0**.**15** ± **0**.**01**	**0**.**12** ± **0**.**00**	**0**.**13** ± **0**.**01**	**0**.**12** ± **0**.**00**	**0**.**15** ± **0**.**01**
Community	**0**.**28** ± **0**.**03**	**0**.**02** ± **0**.**00**	**0**.**07** ± **0**.**01**	**0**.**02** ± **0**.**01**	**0**.**22** ± **0**.**03**
Random	**0**.**15** ± **0**.**01**	**0**.**05** ± **0**.**00**	**0**.**09** ± **0**.**02**	**0**.**04** ± **0**.**00**	**0**.**13** ± **0**.**02**
molfreesolv	**3**.**30** ± **0**.**32**	**3**.**90** ± **0**.**24**	**3**.82 ± 0.31	**3**.67 ± 0.37	**3**.**83** ± **0**.**38**
molesol	**1**.**03** ± **0**.**08**	**1**.**03** ± **0**.**07**	**1**.26 ± 0.16	**1**.13 ± 0.16	**1**.**17** ± **0**.**10**
mollipo	**0**.**93** ± **0**.**03**	**0**.**83** ± **0**.**03**	**0**.92 ± 0.05	**0**.81 ± 0.03	**0**.**94** ± **0**.**02**
molclintox	*0.49* ± *0.03*	**0**.**76** ± **0**.**13**	**0**.**72** ± **0**.**10**	**0**.**76** ± **0**.**18**	**0**.**74** ± **0**.**06**
molbbbp	**0**.**64** ± **0**.**02**	**0**.**64** ± **0**.**02**	*0.61* ± *0.03*	**0**.**62** ± **0**.**02**	**0**.**64** ± **0**.**03**
molbace	**0**.**70** ± **0**.**04**	*0.69* ± *0.07*	*0.68* ± *0.05*	*0.67* ± *0.06*	**0**.**72** ± **0**.**05**
molhiv	**0**.**41** ± **0**.**06**	**0**.**55** ± **0**.**11**	**0**.**48** ± **0**.**13**	**0**.**63** ± **0**.**07**	**0**.**52** ± **0**.**11**

For the classification datasets we report AUROC and for regression datasets RMSE. Bold text indicates a result performs equally (or better) than the model pre-trained on molecules (**Chem**), with Ilalic text indicating the opposite. Molecular tasks include domain features in fine-tuning

When evaluating a family of models over many benchmark tasks, it is not enough to report per-dataset metrics for significance, the overall comparison must be guarded against the inflated Type-I error that arises from multiple pairwise tests. Critical-difference (CD) diagrams, built on the Friedman-Nemenyi framework, meet this need by (i) ranking each model on every dataset, (ii) testing whether the observed ordering could have occurred by chance, and (iii) visualising any groups of models whose average ranks cannot be distinguished at the chosen significance level. In short, the CD diagram is well suited to our ablation analysis of ToP-Social by determining a significant dataset contributing to ToP-Social’s performance.

The critical-difference diagram in Fig. 9 computes the ‘critical difference’ at $p \le 0.05$. The CD bar shows the minimum gap in average ranks required for significance if two models’ rank intervals overlap this bar, they are not significantly different; only gaps exceeding the CD indicate a reliable performance difference.

In our ablation study of ToP-Social, from Table  12 (Fig. 9), no single dataset’s removal produces an average-rank shift exceeding the CD, demonstrating that no non-chemical domain contributes disproportionately to the ToP-Social’s superior transfer performance.

### Noising

Assume some imbalance between features and structure, $I(y;X) \ne I(y;E)$. Here we propose a way of investigating this imbalance between feature information and structure information. By degrading the useful information in either (or both of) *X* and *E*, the degree to which performance relies on one or the other should be apparent. A caveat here is that degrading either will presumably also degrade the information from their interdependence.

Consider some destructive noising process $N_t(\cdot )$, that degrades the useful information in either *X* or *E*. $N_0(x) = x$, with no useful information after $N_T(\cdot )$.

From here edge noising is denoted $N_E = N_t(E)$, and node feature noising $N_X = N_t(X)$. Assume some imperfect model produces predictions $f(X, E) \rightarrow \hat{y}$. Performance is some $h(y, \hat{y})$ between real and predicted values. By sampling $h(y, f(N_X, N_E) \rightarrow \hat{y})$ the level of use of information from both *X* and *E* should be observable. We apply this experiment over our molecular benchmarks. Here *X* includes both node and edge features.

*Structure noise* We use random edge removal/addition for structure noise. At *t*, we remove $|E_r| = p_t \cdot |V|$ edges at random from the graph, $E' = E \setminus E_r$. We then add an equal number of randomly chosen edges $|E_a| = p_t \cdot |V|$, for a final noised edgelist $E'' = E' \cup E_a$.

This is very close to the procedure taken by graph diffusion models as forward noising processes (Vignac et al. 2023). At $N_T(E)$, the structure is a random graph with the same density as the original.

*Feature Noise - Permutation*

We employ additive Bernoulli noise across node features. At *t*, we have a probability of permuting the entries across a feature $p_t$. Let node (or edge features) across a whole dataset be $X \in \{0,1\}^{N \times D}$ with *N* points and *D* features. At *t* we randomly select $N_f = p_t \cdot D$ features to permute. Each selected feature is then shuffled, across the whole dataset, removing the useful information from that feature that pertains to each sample, while maintaining the marginal distribution of the given feature.

*Results* We fix the test set of each benchmark as un-noised, then perform 8 supervised training runs across 8 increasing noise levels on features and structure. In order to reduce cost, and in contrast to our main transfer results, we perform these supervised training runs only for 25 epochs. When noise is applied to structure, no noise is applied to features, and vice-versa. We present the results in Fig. 10 for regression datasets and Fig. 11 for classification datasets.

Performance suffers by varying amounts across different datasets. On most datasets the untrained GIN’s response to structural noise is minimal, with little performance change even at complete noise in the graph structure. The untrained GIN’s reponse to feature noise is far larger, in particular on regression datasets (molesol, molfreesolv, mollipo). The Chem model, pre-trained only on molecules, follows the same patterns as the untrained GIN model.

On most datasets the two non-molecular models, Social and All, show large performance drops as both structural and feature noise increases. This is most clear on the datasets where ToP pre-training was most effective, for example molclintox. Interestingly this suggests that ToP pre-training allows models not only to use structural patterns to greater effect, but also integrate information from features more usefully. An additional possibility is that ToP pre-training protects against overfitting to patterns in graph features, hence those features being used to greater effect.

## Footnotes

1 Galactica is trained on scientific literature, including but not limited to chemistry.

2 Our GCC version, updated to current packages, will be available on GitHub.
