# Supplementary material for: Topology only pre-training: towards generalised multi-domain graph models
Source: Data Min Knowl Discov. 2026 May 9;40(4):44. doi: 10.1007/s10618-026-01210-1 (PMC13157451; doi:10.1007/s10618-026-01210-1)
Supplement: Supplementary file 1 — (pdf 93 KB) [file 10618_2026_1210_MOESM1_ESM.pdf]

Cover Letter for Data Mining and Knowledge Discovery Journal

Summary of Contribution

Main Claim and Importance:

This paper presents Topology Only Pre-Training (ToP), a novel multi-domain pre-training method for graph neural networks that enables transfer learning across diverse graph domains by excluding node and edge features during pre-training while maintaining the ability to reintegrate them during fine-tuning. This represents a fundamental contribution to graph machine learning research by breaking the domain-specificity barrier—ToP is the first approach to demonstrate consistent positive transfer across multiple highly heterogeneous graph domains (molecules, social networks, road networks, neural connectomes) without requiring domain-specific features, LLMs, or feature transformations. Moreover, we demonstrate that diverse out-of-domain pre-training can outperform in-domain pre-training, directly contradicting assumptions pervasive in current literature that multi-domain graph learning shows "no positive transfer benefit." ToP enables transfer learning for data-scarce domains and provides a foundation for graph foundation models, analogous to BERT for text or ImageNet models for vision, but without requiring orders of magnitude more parameters or computational resources.

Evidence Supporting Claims

Our claims are supported by rigorous statistical evidence including 10 independent fine-tuning runs per task across 13 benchmark datasets, with Wilcoxon signed-rank tests ($p \leq 0.01$) demonstrating that 76% of tasks show significant positive transfer versus supervised baselines, increasing to 85.7% when features are included in fine-tuning. We conducted comprehensive ablation studies using critical difference analysis (Friedman-Nemenyi framework) confirming that no single dataset drives performance, with systematic dataset exclusion experiments validating that overall diversity, not individual domains, drives benefits.

Our comparative benchmarks show that ToP outperforms domain-specific baselines on 79% of molecular benchmarks when pre-trained on non-molecular data, achieves competitive performance with LLMs 1000× larger (3B-30B parameters vs. 2.4M), and surpasses GCC—the only comparable generalist GNN approach—across all tasks. We provide mechanistic analysis through PCA correlation analysis with graph-level metrics, iterative noising experiments demonstrating structural bias acquisition, and UMAP visualizations showing learned topological clustering.

Related Work (Past 7 Years, External Groups)

Qiu et al. (2020) presented "GCC: Graph Contrastive Coding" (KDD), the most similar prior work attempting domain-agnostic graph learning through ego-network sampling and positional encodings. ToP differs by enabling feature reintegration downstream, focusing on graph-level tasks, and demonstrating superior performance through adversarial augmentations. Liu et al. (2023) proposed "One For All: Towards Training One Graph Model For All Classification Tasks" (ICLR), using LLM-based feature unification for multi-domain graphs. ToP provides an alternative that requires no auxiliary LLMs, works without text-encodable features, extends to any graph-level task, and achieves competitive performance at a fraction of computational cost.

Hu et al. (2020) established contrastive pre-training for domain-specific molecular graphs in "Strategies for Pre-training Graph Neural Networks" (ICLR). ToP extends this to multi-domain settings and demonstrates that structural learning alone enables cross-domain transfer. Mao et al. (2024) published a position paper "Graph Foundation Models Are Already Here" (ICML) arguing LLMs are necessary for graph foundation models due to assumed lack of multi-domain transfer. Our work empirically refutes this assumption and provides an efficient alternative pathway. Finally, Xu et al. (2024) presented "LLM and GNN are Complementary: Distilling LLM for Multimodal Graph Learning" (arXiv), using LLMs to distill knowledge into GNNs for multi-domain learning. ToP demonstrates multi-domain learning is achievable without LLMs, though LLM integration could be a complementary future direction.

Keywords

General Keywords: Graph Neural Networks, Transfer Learning, Representation Learning, Multi-Domain Learning, Foundation Models
Specific Keywords: Topology-Only Pre-Training, Graph Contrastive Learning, Cross-Domain Graph Transfer, Feature-Agnostic Graph Encoding, Adversarial Graph Augmentation

Conflict of Interest Declaration

We declare institutional conflicts of interest with the University of Bristol (`@bristol.ac.uk`), the current affiliation of all authors.

Suggested Reviewers

Jure Leskovec Co-author of foundational work, Stanford, `jure@cs.stanford.edu`

Marinka Zitnik Leading researcher in GFMs, Harvard, `marinka@hms.harvard.edu`

Michael Galkin Work on GFMs, Google, `mgalkin@google.com`

Bryan Perozzi Work on GFMs, Stonybrook, `bperozzi@cs.stonybrook.edu`

Yuan Fang GFM Surveys, Singapore Management University, `yfang@smu.edu.sg`

We believe this work makes significant contributions to enabling practical transfer learning for graphs and opens new research directions in graph foundation models. We look forward to your consideration.
Sincerely,
Alex O. Davies, Riku Green, Telmo M. Silva Filho, and Nirav Ajmeri
